# Multi-approach integrating water quality variables, remote sensing, eDNA and histopathology for assessing the impact of microbial pollution on Nile Tilapia from Kitchener Drain, Northern Egypt

**DOI:** 10.1186/s12866-026-05559-7

**Published:** 2026-09-05

**Authors:** Muhammad A. El-Alfy, Ahmad M. Alzeny, Ahmed E. Abouelwafa, Engy T. Megahed, Marwa M. AbouHadied, Mohamed M. Toubar, Hazem T. Abd El-Hamid, Mohamed A. H. El-Kady, Hala A. Mansour

**Affiliations:** National Institute of Oceanography and Fisheries, NIOF, Cairo, Egypt

**Keywords:** Fish, eDNA, Remote sensing, Bioinformatics, Microbial pollution

## Abstract

This study integrated environmental DNA (eDNA) metabarcoding, conventional bacteriology, histopathology, water quality variables, and remote sensing indices to investigate how environmental gradients are associated with pathogen-associated taxa communities and influence Nile tilapia (*Oreochromis niloticus*) health in Kitchener Drain, Northern Egypt. Physicochemical analyses revealed elevated nutrient concentrations, chlorophyll-a (59.21–226.35 µg L⁻^1^), with localized hypoxia (dissolved oxygen as low as 2.14 mg L⁻^1^ in site 2). High-throughput 16S rRNA metabarcoding revealed microbial communities dominated by Actinobacteriota (27.35%) and Proteobacteria (26.73%), with beta-diversity patterns driven primarily by species turnover, indicating strong environmental filtering along the pollution gradient. eDNA metabarcoding detected pathogen-associated taxa, including Mycobacterium, Legionella, Burkholderiaceae, Erysipelothrix, Leptospira, and Flavobacterium, many of which were not recovered by culture-based methods. Cross-database integration of SILVA (v138) and GTDB (r214) references identified 256 ASVs assigned to pathogen-associated taxa across 32 genera, with the dual-database strategy detecting 20–52% additional pathogen-associated ASVs relative to either database alone. Spearman correlation was examined for the 27 taxa with physicochemical parameters; phosphate, chlorophyll-a, dissolved oxygen, and BOD came up most often. Spearman correlation analysis revealed two perfect taxon–environment associations: a positive correlation between G_Pseudomonas and nitrate (ρ = + 1.00) and a negative correlation between G_Acinetobacter and chlorophyll-a (ρ = − 1.00). Both associations were nominally significant before multiple-testing correction (uncorrected p = 0.017); however, neither remained statistically significant after correction for multiple comparisons. These findings were therefore interpreted as exploratory associations rather than evidence of environmental drivers or causal relationships. Integration of remote sensing indices (NDWI, NDTI, NDCI, CDOM, NDVI) and water variables aids in identification of the surrounding environment associated with the presence of species. Culture-dependent analyses identified *Aeromonas* spp. as the dominant intestinal isolate and the only genus consistently observed by both approaches, suggesting selective host filtering between environmental and gut-associated communities. Histopathological examination revealed epithelial necrosis, villous atrophy, and goblet cell hyperplasia, with the most severe lesions at sites of highest organic loading. This approach can aid as a database for further future work, and for establishing more advanced models of pathogen-associated eDNA distributions and targeted fish-health surveillance, and for maintaining biodiversity.

**Supplementary Information:**

The online version contains supplementary material available at https://doi.org/10.1186/s12866-026-05559-7.

## Introduction

Freshwater ecosystems are increasingly threatened by anthropogenic pollution resulting from agricultural runoff, industrial discharge, urbanization, and untreated wastewater inputs. These environmental stressors contribute to eutrophication, nutrient enrichment, oxygen depletion, and deterioration of water quality, ultimately affecting aquatic biodiversity and fish health [1, 2].

In Egypt, drainage systems such as Kitchener Drain receive large quantities of domestic, agricultural, and industrial effluents that negatively influence water quality and create favorable conditions for microbial proliferation and pathogen dissemination [3]. Such polluted aquatic environments may act as reservoirs for opportunistic and zoonotic microorganisms capable of threatening aquaculture sustainability and public health. Nile tilapia (*Oreochromis niloticus*) is one of the most economically important freshwater fish species worldwide and represents a major component of Egyptian aquaculture production [4, 5]. However, deteriorating environmental conditions increase fish susceptibility to microbial infections and disease outbreaks. Bacterial pathogens such as Aeromonas spp., Salmonella spp., Vibrio spp., Pseudomonas spp., and Enterococcus spp. are frequently associated with diseased freshwater fish and may cause severe economic losses in aquaculture systems, while posing health risks to consumers [6, 8].

Evaluating ecosystem health and microbial pollution along such impacted fresh water bodies requires an integrated multi-tiered approach. Traditional bacteriological methods remain essential for detecting viable pathogens and confirming active microbial growth. However, culture-dependent approaches often underestimate microbial diversity because many aquatic microorganisms are slow-growing, fastidious, or viable-but-non-culturable (VBNC) under laboratory conditions [9, 10]. To overcome these limitations, environmental DNA (eDNA) metabarcoding has emerged as a powerful molecular tool for investigating microbial communities and detecting DNA from uncultured and low-abundance microbial taxa in aquatic ecosystems [11, 12].

High-throughput sequencing combined with bioinformatics pipelines such as QIIME2 and DADA2 enables high-resolution analysis of microbial communities through amplicon sequence variants (ASVs), improving taxonomic resolution and ecological interpretation [13, 14]. In parallel, remote sensing technologies have also become valuable tools for monitoring environmental conditions and water quality in freshwater ecosystems. Satellite-derived spectral indices, including the Normalized Difference Chlorophyll Index (NDCI), Normalized Difference Water Index (NDWI), and Colored Dissolved Organic Matter (CDOM), can provide spatial information related to nutrient enrichment, suspended organic matter, chlorophyll concentration, and eutrophication status [15, 17]. Furthermore, histopathological examinations of target organs, such as fish intestine provide biological evidence of environmental stress and microbial exposure. Lesions such as epithelial necrosis, inflammatory infiltration, goblet cell hyperplasia, and villous degeneration are commonly associated with bacterial enteritis and chronic exposure to polluted aquatic environments [18].

Recent studies suggested that remotely sensed environmental variables can be integrated with microbial datasets to support pathogen prediction and aquatic disease surveillance [19, 21]. Few studies in Egyptian drainage systems have combined eDNA metabarcoding with satellite-derived spectral indices, culture-based bacteriology, and histopathological fish health assessment to characterize associations between environmental gradients, eDNA detection patterns of pathogen-associated taxa, and biological responses along a continuous pollution gradient.

The key innovation of this study lies in integrating these complementary diagnostic frameworks into a unified assessment of ecosystem health. Rather than analyzing these tools separately, this work links satellite-derived environmental indicators, molecular and culture-based microbial monitoring, and direct histopathological evidence of host health deterioration at the drainage system level.

We hypothesized that nutrient enrichment, organic pollution, and environmental gradients detected through both physicochemical measurements and remote sensing indices act as major drivers of microbial community assembly and pathogen occurrence in Kitchener Drain. We further hypothesized that pathogen-associated taxa detected in environmental DNA would exhibit site-specific relationships with environmental conditions and that locations showing greater microbial pollution would be associated with more pronounced intestinal pathological alterations in Nile tilapia.

Therefore, the present study aimed to investigate the ecological relationships among water quality, remotely sensed environmental indicators, aquatic microbial communities, pathogen-associated taxa, and fish intestinal health along Kitchener Drain. To achieve this objective, environmental DNA metabarcoding was integrated with conventional bacteriological analysis, remote sensing-derived spectral indices, and histopathological examination of Nile tilapia.

## Materials and methods

### Study area

Kitchener drain is one of the longest and most heavily polluted agricultural drains in Egypt. It covers three governorates: Dakahlia, Gharbia, and Kafr El-Sheikh. It discharges into the Mediterranean Sea [22]. The drain was designed to manage the agricultural runoff and wastewater. Over time, it receives untreated municipal, industrial and agricultural effluents leading to severe water quality degradation [23]. The water quality in Kitchener drain is affected by high concentrations of pollutants including heavy metals and pesticides that cause adverse effects on aquatic life and human health [3].

**Fig. 1:**
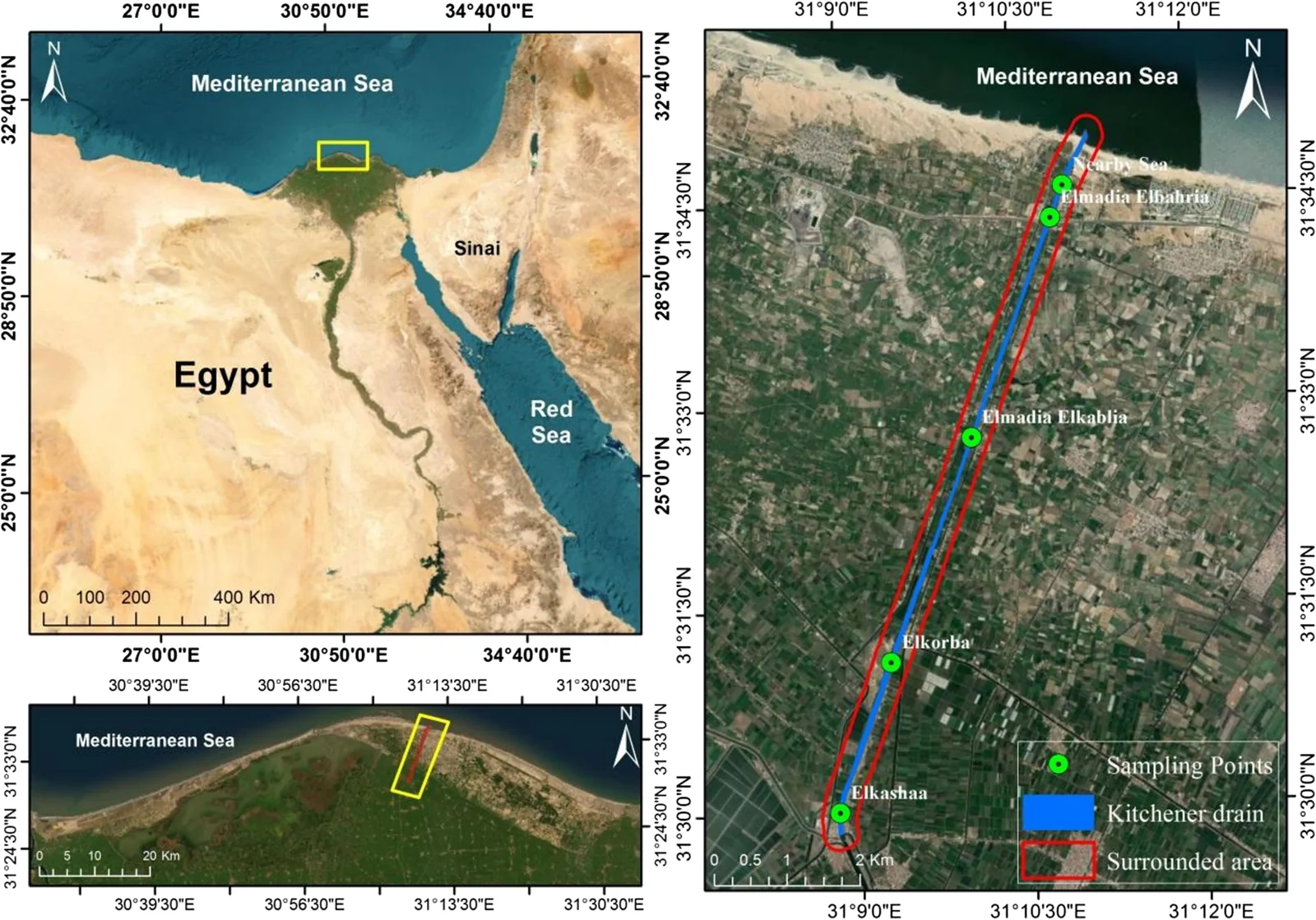
Sampling sites along the studied area of Kitchener Drain. Source of background satellite image: ESRI, Maxar, Earthstar Geographics, and the GIS user. The map was extracted using ArcGIS 10.5

The drain is frequently used for irrigation despite its pollution, making it a key site for assessing the impacts of chemical and microbial contaminants on freshwater ecosystems and aquaculture productivity. The local population depends on the water of Kitchener drain for irrigation and agricultural activities [1]. The five sampling sites span a continuous pollution gradient from the Mediterranean-proximal estuary (Site 1, Nearby Sea) to inland agricultural discharge points (Site 5, Elkashaa), providing a natural framework for linking environmental gradients to microbial community responses and fish health outcomes. The locations and coordinates were as shown in Table 1 and Fig. 1.

**Table 1:** Coordinates of sampling sites and description

No	Name & description	Latitude (N)	Longitude (E)
1	Nearby Sea	31.576475	31.182216
2	Elmadia Elbahria	31.572525	31.180252
3	Elmadia Elkablia	31.545697	31.167747
4	Elkorba	31.518272	31.154975
5	Elkashaa	31.499916	31.146822

### Water samples collection and analyses

Water, sediment, and fish samples were collected on 24 June 2025 from five sites along the northern part of Kitchener Drain. The samples were preserved for transportation to the laboratory. The water samples were analyzed for determination of different parameters. Depth and temperature were measured in the field. Salinity, EC and total dissolved solids were measured using salinity-meter. Dissolved oxygen (DO), and biological oxygen demand (BOD) were analyzed according to methods of APHA [24]. Nutrients (NH_4_, NO_2_, NO_3_, PO_4_, SiO_4_, TN and TP) were analyzed according to the methods of Grasshoff et al. [25]. Chlorophyll a was determined in the samples based on Strickland and Parsons method [26]. The oxidizable organic matter (OOM) in water was assessed using FAO method [27].

### Fish samples collection and preparation for traditional bacterial isolation analysis

Three Nile tilapia specimens were collected from each site (n = 15 total). Intestinal samples of *Oreochromis niloticus* were collected aseptically and homogenized in sterile physiological saline (0.85% NaCl), followed by serial dilution for bacteriological analyses. Thermotolerant coliforms (*Escherichia coli*) were enumerated on m-FC agar at 44.5 °C for 24 h. Selective media were used for the isolation of pathogenic bacteria, including Aeromonas selective agar with ampicillin for *Aeromonas* spp., Mannitol Salt Agar for *Staphylococcus* spp., TCBS agar for *Vibrio* spp., Bismuth Sulphite Agar for *Salmonella* spp., Slanetz and Bartley medium for *Enterococcus* spp., and cetrimide-based Pseudomonas agar for *Pseudomonas* spp. Plates were incubated at 37 °C for 48 h unless otherwise stated. All analyses were conducted in triplicate, and bacterial counts were expressed as CFU g⁻^1^ (mean ± SD) following Austin and Austin [6] and Hassan et al. [28].

### Extraction of environmental DNA (eDNA)

Water samples were filtered through 0.45 μm cellulose nitrate membrane filters for eDNA extraction. As a volume of about 1645, 1215, 900, 825 and 1660 ml from water was filtered for sites from 1 to 5, respectively. The filtration occurred through 2 to 3 filter papers for water samples. This filtration process was done according to the nature of the samples and clogging process, so the filtration occurred with no equal volumes. Then preservation of filters was done in absolute ethyl alcohol until further use. Genomic DNA was extracted from the preserved filter papers using ABT genomic DNA mini extraction kit (ABT, EGYPT) according to the manufacturer protocol. Field blanks, filtration blanks, extraction blanks, and no-template PCR controls were not included in this study. For amplicon library construction of the 16S rRNA (V3-V4) genes, samples were prepared according to the 16S Metagenomic Sequencing Library protocols by Macrogen Ltd (Seoul, Korea). The barcoded primer sequences were: Bakt_341F (5′-CCTACGGGNGGCWGCAG-3′) and Bakt_805R (5′-GACTACHVGGGTATCTAATCC-3′). Sequencing was performed as single-end reads. Although the V3–V4 region is commonly sequenced using paired-end chemistry, single-end sequencing was used in the present study according to the sequencing service/provider workflow and the downstream bioinformatics pipeline was adjusted accordingly.

### Bioinformatics

Raw single-end 16S rRNA gene sequences were processed using QIIME 2 (v2024.5.1), in a Conda-based WSL2 environment [14]. Primer trimming and quality filtering were performed using Cutadapt [29], while amplicon sequence variants (ASVs) were inferred using DADA2 [13] after sequence truncation at 280 bp. Taxonomic classification was performed independently against SILVA v138 and GTDB r214-. For SILVA, the pre-trained silva-138–99-nb-classifier.qza, based on 99% reference sequence clustering, was applied with q2-feature-classifier classify-sklearn using automatic read-orientation detection and the confidence threshold of 0.70; the SILVA classifier was not retrained locally [30, 31]. For the GTDB comparison, a region-specific Naive Bayes classifier was generated from GTDB r214 16S rRNA reference sequences after 99% dereplication and in silico extraction of the V3-V4 region with the study primers. Reference amplicons between 250 and 500 bp were retained, and GTDB assignments were accepted at a confidence threshold of 0.70 [32].

Pathogen-associated taxa were designated using a predefined filtering procedure in R. Unassigned sequences and ASVs classified as chloroplasts, mitochondria, Eukaryota, or other non-bacterial lineages were excluded. SILVA and GTDB assignments were integrated by ASV identifier; concordant assignments were counted once, the more resolved compatible assignment was retained, and conflicting assignments were excluded.

Downstream community analysis was performed in R version 4.4.3 [33] with phyloseq [34]. Data visualizations were generated in R using the ggplot2 package [35]. After restricting the data to Bacteria and removing chloroplast and mitochondrial assignments, relative-abundance barplots were generated at phylum and genus levels; lower-abundance taxa were grouped as “Other” to retain approximately 95% and 90% mean abundance coverage, respectively. For alpha diversity, the cleaned community object was rarefied to the minimum post-denoising library size (72,103 reads). Observed ASV richness, Shannon diversity, and Simpson diversity were then calculated. Rarefaction curves were generated from the ASV count matrix with vegan::rarefy across a depth grid extending to the common minimum depth. Bray–Curtis distances and PCoA were calculated with phyloseq::distance(method = 'bray') and ordinate(method = 'PCoA'), respectively. Presence-absence beta diversity was partitioned with betapart::beta.pair(index.family = 'sorensen') into total Sørensen dissimilarity (beta.sor), turnover (beta.sim), and nestedness (beta.sne) components [36]. The heatmap included the 50 genera with the highest mean relative abundance after taxonomic agglomeration, log_10_(relative abundance + 10⁻⁶) transformation, and row-wise Z-score normalization. Representative sequences were aligned with MAFFT [37], phylogenetic trees were generated with FastTree [38] and trees were visualized in iTOL [39].

### Extracted remotely sensing spectral indices

An image of Sentinel S2C_MSI_L2A sensor was downloaded from https://browser.dataspace.copernicus.eu/. The acquisition date of the satellite image was the same as the sampling date at 24/6/2025. The cloud coverage assessment in the metadata file of the image was > 8.684637. The resampling technique for band 5 using nearest resampling technique of Arc Toolbox in ArcGIS 10.5 program as follows (Arc Toolbox, Data Management Tools, Raster, Raster Processing, and then Resampling Technique of Nearest). The calculated spectral indices from Sentinel S2C L2A sensor were applied using Rater Calculator Tool in Arc Toolbox of ArcGIS 10.5 (Arc Toolbox, Spatial Analyst Tools, Map Algebra, and then Raster Calculator) were applied according to formula in Table 2. For extraction of pixel values at each site a buffer of 10 m around each site was done and random points of ten were selected. Then the extraction process occurred for further statistical analyses and correlation with pathogen-associated taxa and other microbial taxa. The buffer was established as follows (Arc Toolbox, Proximity and then Buffer). For random points (Arc Toolbox, Data Management Tools, Sampling, Create Random Points, Number of Points; Long = 10). Finally the extraction occurred as follows; (Arc Toolbox, Spatial Analyst Tools, Extraction, and then Extraction Values to Points for further statistical analyses within field and lab data).

**Table 2:** The spectral indices applied in the studied area of Kitchener drain

Index	Sensor	Formula	Purpose/Use	Reference
Normalized difference vegetation index (NDVI)	Sentinel 2 C L2A	NIR-Red/NIR + Red	Vegetation cover and density	Tucker [40]
Normalized difference water index (NDWI)		Green-NIR/Green + NIR	Water surfaces and wetlands	Avezov et al. [17]
Normalized difference turbidity index (NDTI)		Red-Green/Red + Green	Turbidity and suspended matters	Lacaux et al. [41]
Normalized difference chlorophyll index (NDCI)		RedEdge—Red/RedEdge + Red	Chlorophyll indication	Mishra and Mishra [16]
Colored dissolved organic matter (CDOM)		RedEdge/Blue	Dissolved organic matters	Shang et al. [42]

NIR: near infrared (B8, resolution = 10 m), Red (B4, resolution = 10 m), Green (B3, resolution = 10 m), RedEdge (B5, resolution = 20 m), Blue (B2, resolution = 10 m). Resampling was applied to B5

### Histological examination

#### Fish sampling and tissue preservation

A total of 15 Nile tilapia (*Oreochromis niloticus*) specimens (n = 3 fish per site) were collected from five sampling locations immediately following euthanasia, a 1 cm segment of the anterior intestine, approximately 2 cm posterior to the pyloric caeca, was excised using sterile dissecting instruments. The intestinal tissues were gently rinsed with sterile phosphate-buffered saline (PBS, pH 7.4) to remove intestinal contents and fixed in 10% neutral-buffered formalin (NBF; 4% formaldehyde) for 24–48 h at room temperature. After fixation, tissues were transferred to 70% ethanol until histological processing [43].

#### Histological processing and slide preparation

Following fixation, the intestinal tissues were processed routinely by dehydration in graded ethanol, clearing in xylene, and embedding in paraffin wax according to Bancroft and Gamble [43]. Serial cross-sections were cut at 4.5 µm using a rotary microtome. For general histological screening, three non-consecutive cross-sections per fish were prepared (9 slides per site; 45 slides in total) and stained with Hematoxylin and Eosin (H&E).

#### Blind evaluation

To reduce observer bias, all slides were coded with random numerical identifiers before microscopic examination. Histopathological evaluation was performed without knowledge of the corresponding sampling site.

#### Microscopic evaluation and Field-of-View (FOV) criteria

Each fish was represented by three histological sections. Ten non-overlapping fields of view (FOVs) were examined from each section under 200 × and 400 × magnifications, giving a total of 30 FOVs per fish, 90 FOVs per site, and 450 FOVs for the entire study. Examination of H&E-stained sections included the luminal epithelium, mucosal and submucosal layers, and muscularis. Modified Gram-stained sections were assessed for bacterial colonization together with associated inflammatory changes.

#### Modified gram staining

Modified Gram staining was performed to demonstrate Gram-negative bacterial colonization within intestinal tissues. Only representative H&E-stained sections showing mucosal necrosis, inflammatory cell infiltration, or marked structural damage during the initial microscopic examination were selected for Gram staining. Three representative slides from each sampling site were stained, giving a total of 15 slides.

#### Lesion scoring system

Histopathological lesions were evaluated using the semi-quantitative scoring system proposed by Bernet et al. [44]. Histopathological changes were evaluated using a semi-quantitative five-point scoring system (0–4). A score of 0 indicated normal tissue without detectable lesions, whereas scores of 1, 2, 3, and 4 indicated mild, moderate, marked, and severe changes, respectively. The evaluated parameters included epithelial necrosis, inflammatory cell infiltration, goblet cell hyperplasia, and villous atrophy/degeneration [18].

For each fish, lesion scores were assigned for all evaluated parameters, and the mean lesion score was calculated from the examined sections. Site-specific values were expressed as mean ± standard deviation (SD) using the three examined fish per sampling site (n = 3). A Mean Site Index was calculated as the average lesion score for each sampling site.

Histological observations were performed using an Olympus BX43 light microscope (Olympus Corporation, Japan), and representative photomicrographs were captured using an Olympus DP27 digital camera coupled with CellSens Dimensions software (version 1.18).

### Statistical analyses

Multivariate relationships among environmental variables, remotely sensed spectral indices, and the relative abundances of microbial taxa were evaluated using Principal Component Analysis (PCA). The PCA was conducted using the PAST program. To assess the specific relationships between pathogen-associated taxa and physicochemical parameters across the five sampling locations (n = 5), a two-tailed Spearman's rank correlation analysis was performed using Graph Prism 10 [45]. Given the sample size constraints (n = 5), a critical correlation threshold of |r|≥ 0.900 was required to determine statistical significance at p < 0.05 [46, 47]. Consequently, correlation coefficients falling below this threshold (|r|< 0.900) were interpreted conservatively as non-significant ecological associations rather than statistically significant drivers.

## Results

### Characterization of water quality in Kitchener Drain

Physicochemical analysis revealed slightly alkaline and moderately saline water conditions, with pH ranging from 8.12 to 8.37 and salinity from 2 to 4 ppt. Dissolved oxygen concentrations varied between 2.14 and 6.74 mg/L, with the lowest value falling below the permissible Egyptian limit for aquaculture waterways (5 mg/L; [48]). Nutrient concentrations were elevated across all sites, including ammonium (180–1026 µg/L), nitrate (907.41–1358.05 µg/L), and total nitrogen (8771.28–10,613.12 µg/L). Chlorophyll-a concentrations ranged from 59.21 to 226.35 µg/L. The results showed variance between data from down and upstream sites in the northern part of Kitchener drain (Table 3).

**Table 3:** Characteristics of water quality parameters in studied Kitchener drain

No	EC	TDS	Sal	pH	Temp	Depth	DO	OOM	BOD	PO_4_	NH_4_	NO_2_	NO_3_	SiO_4_	TN	TP	Chl
	ms	mg/L	Ppt		◦C	m	mg/L				µg/L						
1	4.076	2720	2	8.12	28.5	3	2.14	4	19.8	21.44	180	327.1888	1358.05	2354	9247.84	130.2	226.35
2	4.368	2908	4	8.21	29	2.5	6.74	8	14.7	20.21	652	370.0424	1160.94	2473	10,613.12	138.88	70.84
3	4.456	2972	3	8.33	28.9	2.75	6.09	4	18	17.46	774	306.544	1047	2375	9318.68	125.86	59.21
4	4.4	2940	3	8.3	28.5	2.25	5.65	12	19.2	17.46	748	253.9936	1003.96	2381	9492.56	134.54	75.1
5	5.62	3750	3	8.37	27	nd	6.74	8	18.3	18.07	1026	229.5952	907.41	2510	8771.28	149.73	82.9
Min	4.076	2720	2	8.12	27	2.25	2.14	4	14.7	17.46	180	229.5952	907.41	2354	8771.28	125.86	59.21
Max	5.62	3750	4	8.37	29	3	6.74	12	19.8	21.44	1026	370.0424	1358.05	2510	10,613.12	149.73	226.35
Av	4.66	3108.57	3	8.26	28.27	2.63	5.18	7.43	17.79	19.08	655.14	298.14	1106.12	2422.43	9546.84	136.40	114.28

*Av.* Average, *Temp.* Temperature, *nd* non-detected, *Chl* chlorophyll

### Application of remotely sensed spectral indices

**Fig. 2:**
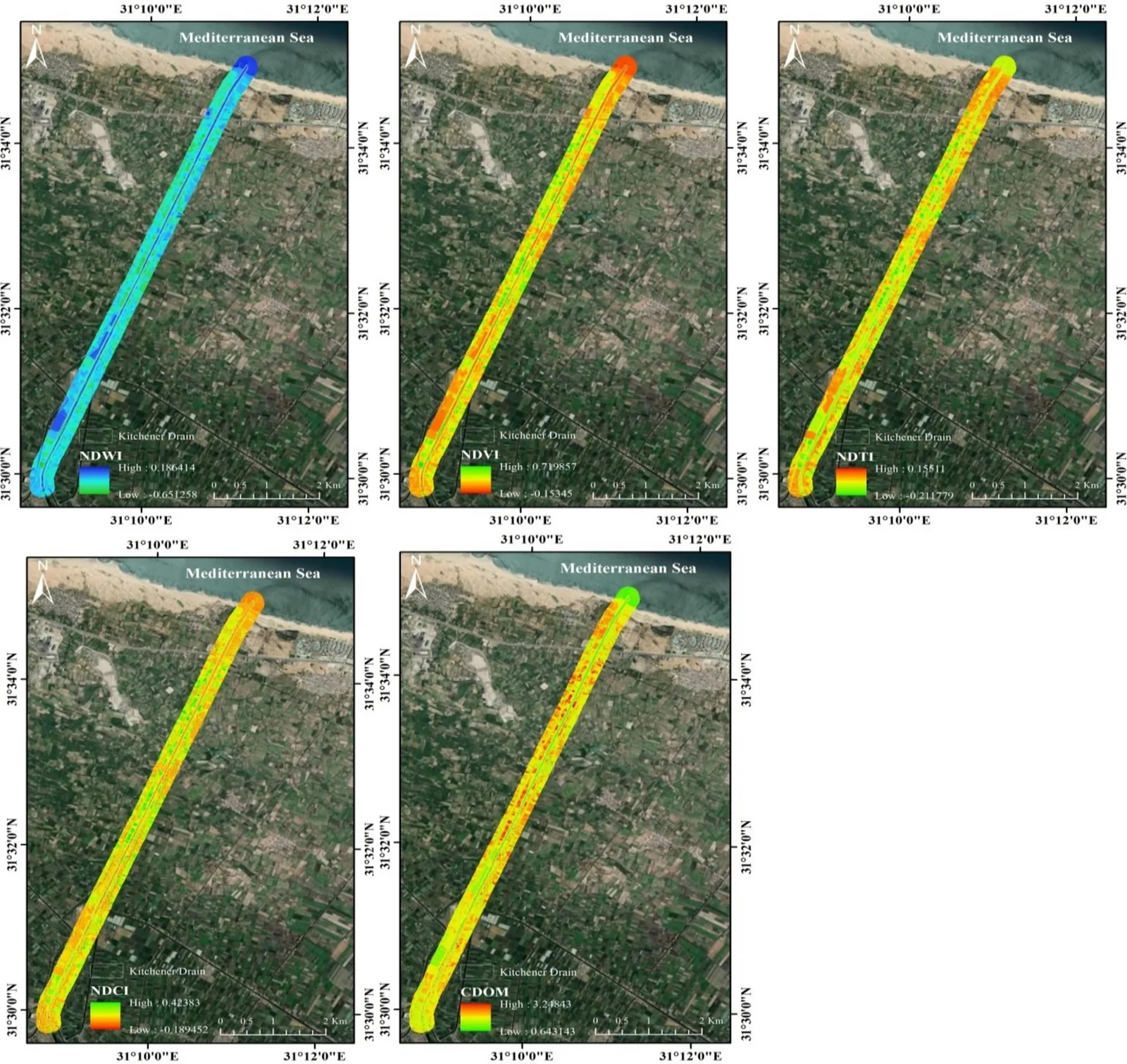
Spectral indices along the Kitchener Drain and in the surrounding area

The values of spectral indices along the study area of Kitchener Drain showed spatial variability in environmental conditions. CDOM varied from 0.634 to 3.248, NDCI from − 0.189 to 0.423, NDTI from − 0.211 to 0.155, NDVI from − 0.153 to 0.719, and NDWI from − 0.651 to 0.186. Integration of spectral indices with satellite data indicated that areas with higher organic enrichment, reflected by elevated OOM (4–12 mg/L) and chlorophyll-a (59.21–226.35 µg/L). The spatial distribution of spectral indices is shown in Fig. 2. These indices were applied in addition to water quality parameters to discover which are more associated with the presence of pathogens. The relative abundance of pathogen-associated taxa was as illustrated in supplementary material (Table S1). The extracted values of spectral indices were also shown in supplementary material (Table S2).

### Bioinformatics analysis of aquatic microbial communities and eDNA diversity

**Fig. 3:**
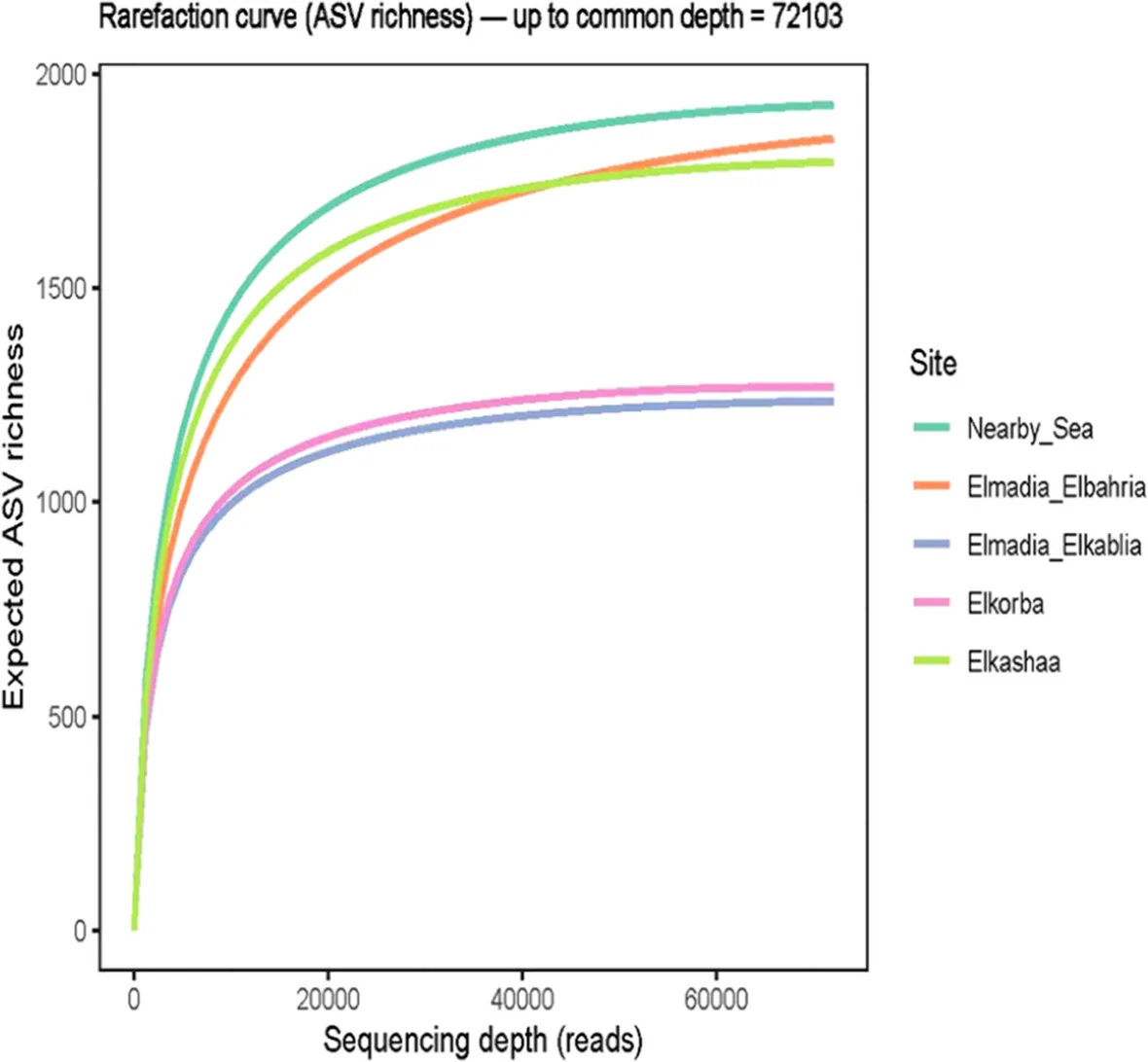
Rarefaction analysis of ASV richness on sampling sites. Rarefaction curves of the expected ASV richness versus sequencing depth across the five sampling sites in the Kitchener Drain. All the samples were randomized to the same depth of 72,103 reads

Rarefaction analysis of 16S rRNA gene sequences processed through QIIME2 and the DADA2 pipeline indicated that all samples reached a clear plateau at a sequencing depth of 72,103 reads, confirming sufficient sequencing coverage and comprehensive representation of microbial diversity across the five sampling sites (Fig. 3).

**Fig. 4:**
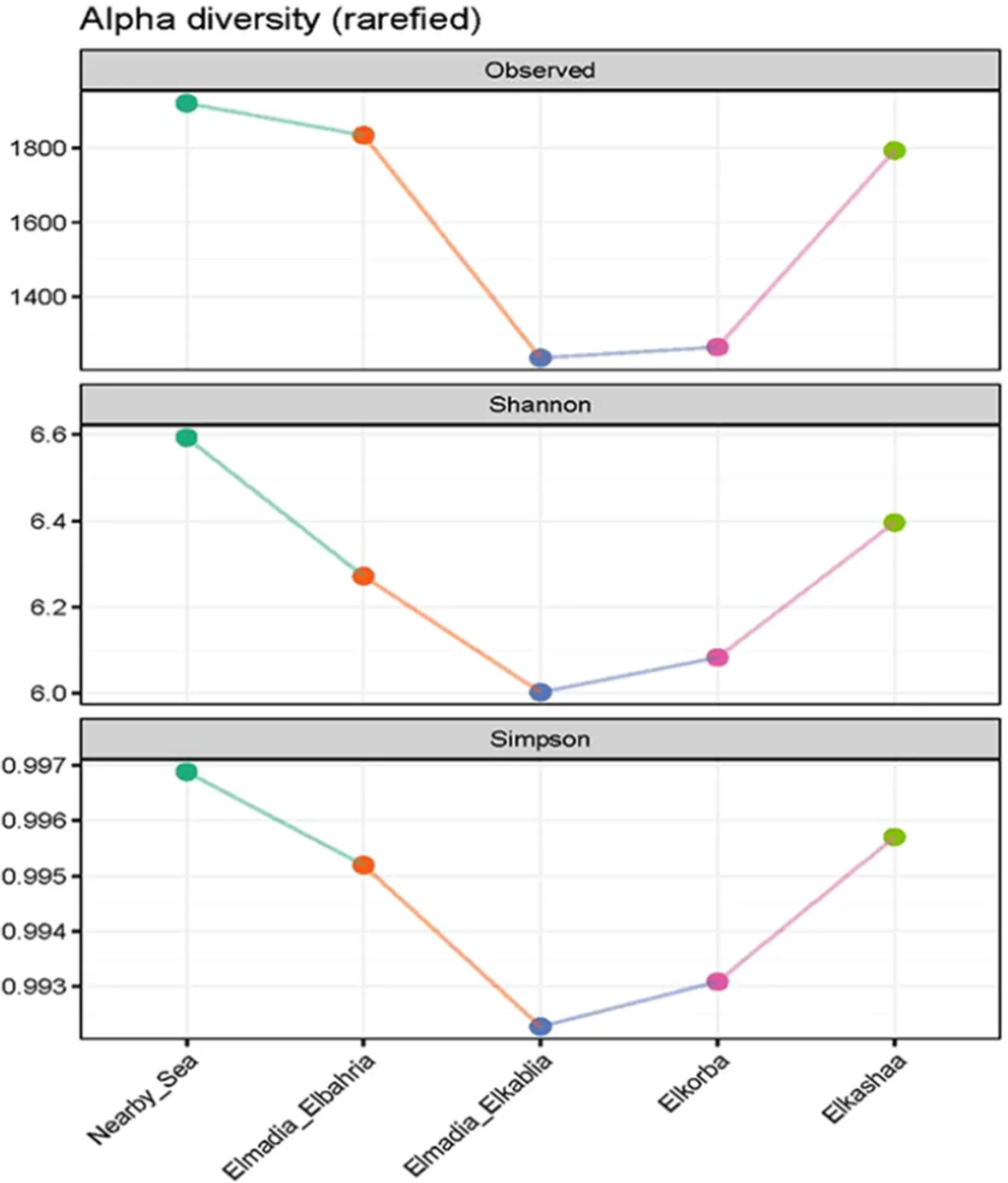
Alpha diversity metrics of the microbial communities on the Kitchener Drain. Diversity in samples within a sample is shown on the panel using **A** the richness of the observed ASV, **B** the Shannon index, and **C** the Simpson index. The major variation is found in the marine-proximal Nearby Sea site, and the lowest values are in the Elmadia Elkablia site

Alpha diversity analysis revealed different gradients of microbial evenness and richness across the drain (Fig. 4). Observed alpha diversity ranged from 1,236 at Elmadia Elkablia to 1,920 at the Nearby Sea site. The Shannon diversity index was highest at the Nearby Sea (6.59) and lowest at Elmadia Elkablia (6) (Fig. 4). The Simpson index showed consistently high evenness across sampling sites (0.992–0.997), even though there are site-specific differences.

**Fig. 5:**
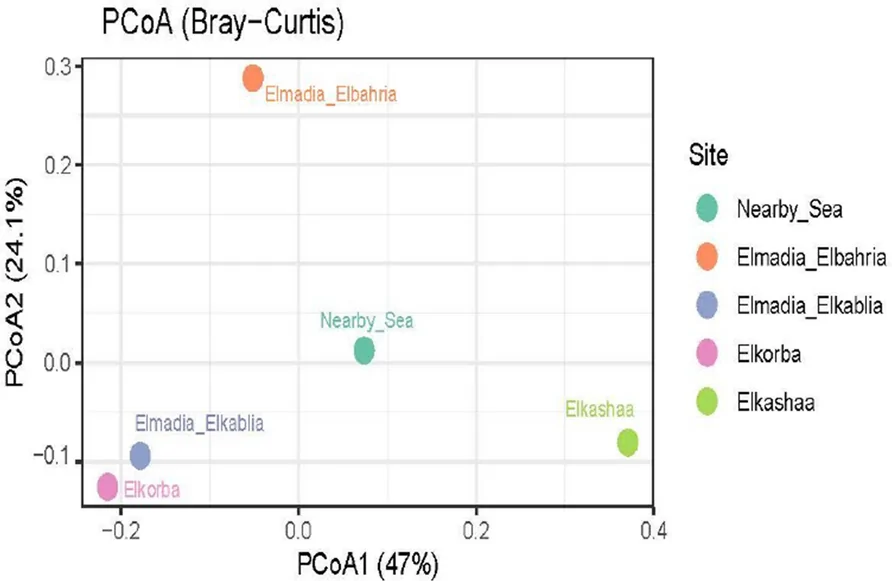
Beta diversity illustrated using Principal Coordinates Analysis (PCoA) using Bray–Curtis dissimilarity. The ordination plot brings out the compositional relationship among the five sites of sampling. The more similar microbial communities, e.g., "Elmadia_Elkablia" and "Elkorba", are represented by points that are closer together, whereas further apart points represent more clustering tendencies and species turnover

Beta diversity analysis based on Bray–Curtis dissimilarity and Principal Coordinates Analysis (PCoA) revealed a spatial variation in microbial community composition among sampling sites (Fig. 5). Elmadia Elkablia and Elkorba showed the greatest similarity, exhibiting the lowest dissimilarity index (0.287), whereas the highest compositional variation was observed between Elkorba and Elkashaa, with a dissimilarity index of 0.604 (Fig. 5).

**Fig. 6:**
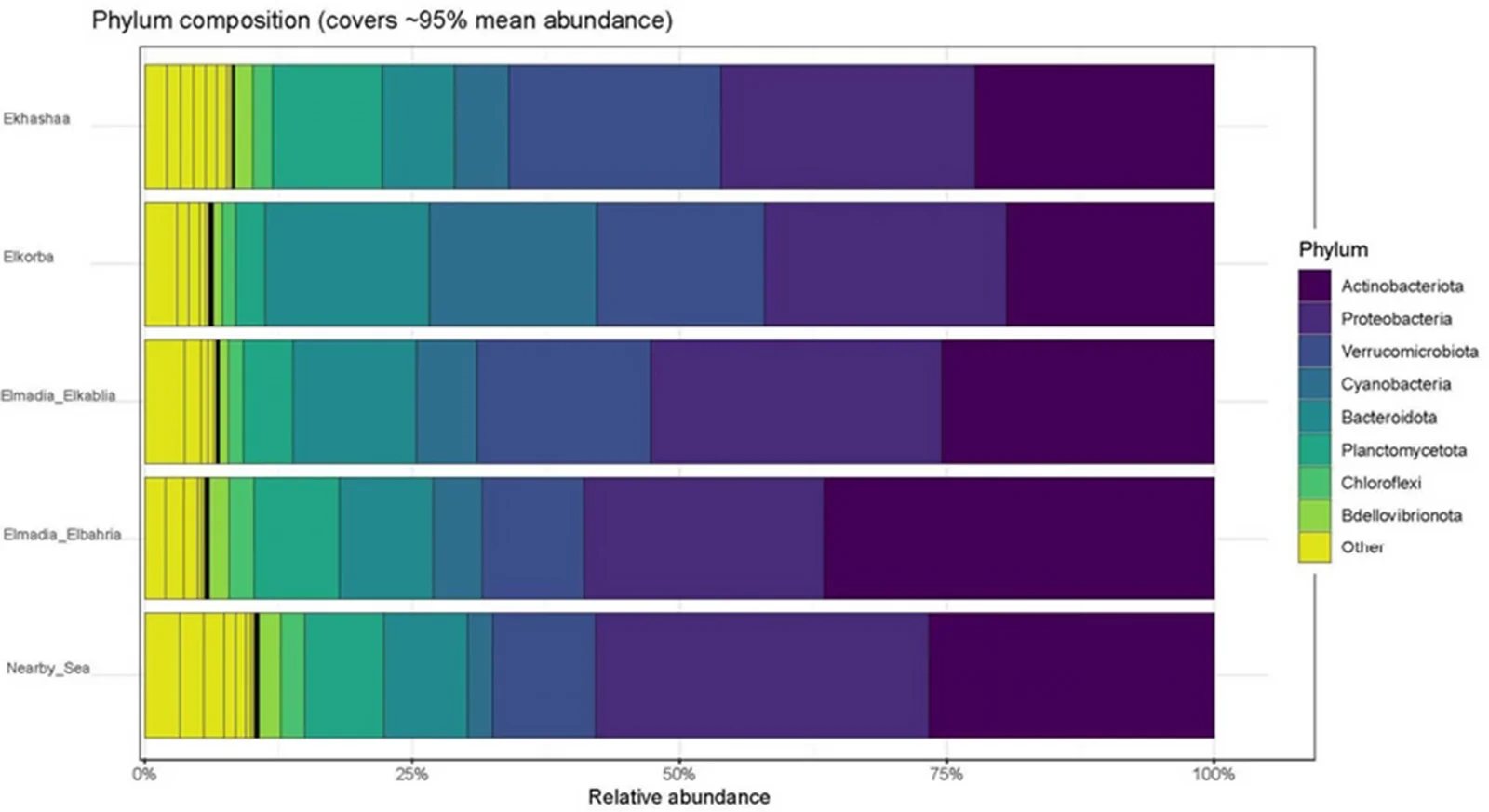
Relative abundance of major bacterial phyla in the five sampling sites in the Kitchener Drain. The bar plot of the taxonomic distribution shows the distribution of more common phyla, with the most predominant one being Actinobacteriota (mean 27.35% ± 6.19) and Proteobacteria (mean 26.73% ± 3.24). This is followed by other dominant groups that are Verrucomicrobiota (15.02% ± 4.96%), Bacteroidota (10.75% ± 4.30%), and Planctomycetota (6.89% ± 2.93%). Compositional differences can be observed on either side of the gradient of the marine-proximal NearbySea site to the inland Elkashaa site

The beta-diversity partitioning based on presence–absence dissimilarity showed that the same site pair had the lowest partitioned dissimilarity value (total beta diversity = 0.456). This partitioned value was mainly explained by species turnover rather than nestedness. Across Kitchener Drain, turnover values ranged from 0.401 to 0.573 and were consistently higher than nestedness values, which ranged from 0.004 to 0.126. The highest partitioned beta-diversity dissimilarity was observed between Elmadia Elkablia and Elkashaa (total beta diversity = 0.636). Notably, variation between the Nearby Sea site and Elmadia Elbahria was mainly explained by turnover (0.573) rather than nestedness (0.004). Microbial communities were dominated by Actinobacteriota (27.35% ± 6.19%) and Proteobacteria (26.73% ± 3.24%), followed by Verrucomicrobiota (15.02% ± 4.96%), Bacteroidota (10.75% ± 4.30%), and Planctomycetota (6.89% ± 2.93%) (Fig. 6). Actinobacteriota reached its highest abundance at Elmadia Elbahria (37.87%), whereas Verrucomicrobiota peaked at Elkashaa (20.61%) (Fig. 6).

**Fig. 7:**
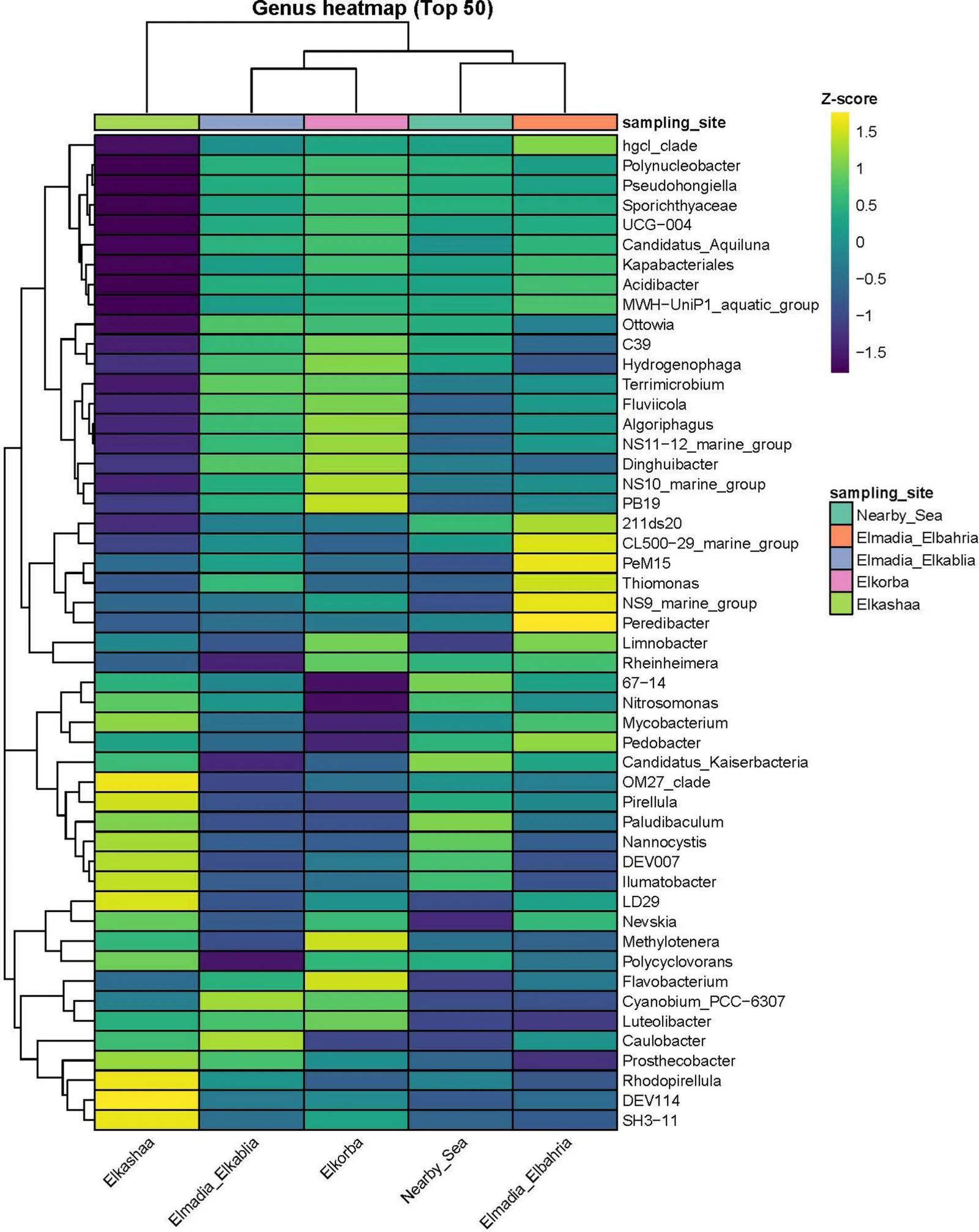
Heatmap of the 50 most abundant bacterial genera. Z-score normalized relative abundances are shown on the heatmap, where yellow color shows enrichment and purple color shows reduction. Hierarchical clustering (top dendrogram) indicates site-specific structuring of the community, with some major groups, such as ElmadiaElkablia and Elkorba, having similar profiles

Z-score normalized heatmap analysis of the top 50 bacterial genera revealed distinct site-specific enrichment patterns across sampling locations (Fig. 7). Cluster analysis showed close similarity between Elmadia Elkablia and Elkorba, where several genera were highly enriched, including *Flavobacterium* (Z-score 0.47 and 1.48), *Algoriphagus* (0.65 and 1.17), *Dinghuibacter* (0.81 and 1.22), and *Hydrogenophaga* (0.68 and 1.09). The Nearby Sea site formed a separate cluster characterized by enrichment of *Candidatus Kaiserbacteria* (1.09), 67–14 (1.02), *Paludibaculum* (1.07), and *Nannocystis* (0.89), while inland-associated genera such as *Flavobacterium* were reduced. Elmadia Elbahria showed enrichment of marine-associated taxa, including NS9marinegroup (1.62), CL500-29marinegroup (1.55), *Peredibacter* (1.75), and PeM15 (1.65), whereas *Pedobacter* reached its highest abundance (1.16). In contrast, Elkashaa exhibited a distinct microbial profile enriched with *Mycobacterium* (1.14), *Nitrosomonas* (0.87), DEV114 (1.73), *Ilumatobacter* (1.42), and OM27clade (1.66), while common aquatic taxa such as hgcI_clade (−1.62) and Polynucleobacter (−1.76) were markedly depleted (Fig. 7).

**Fig. 8:**
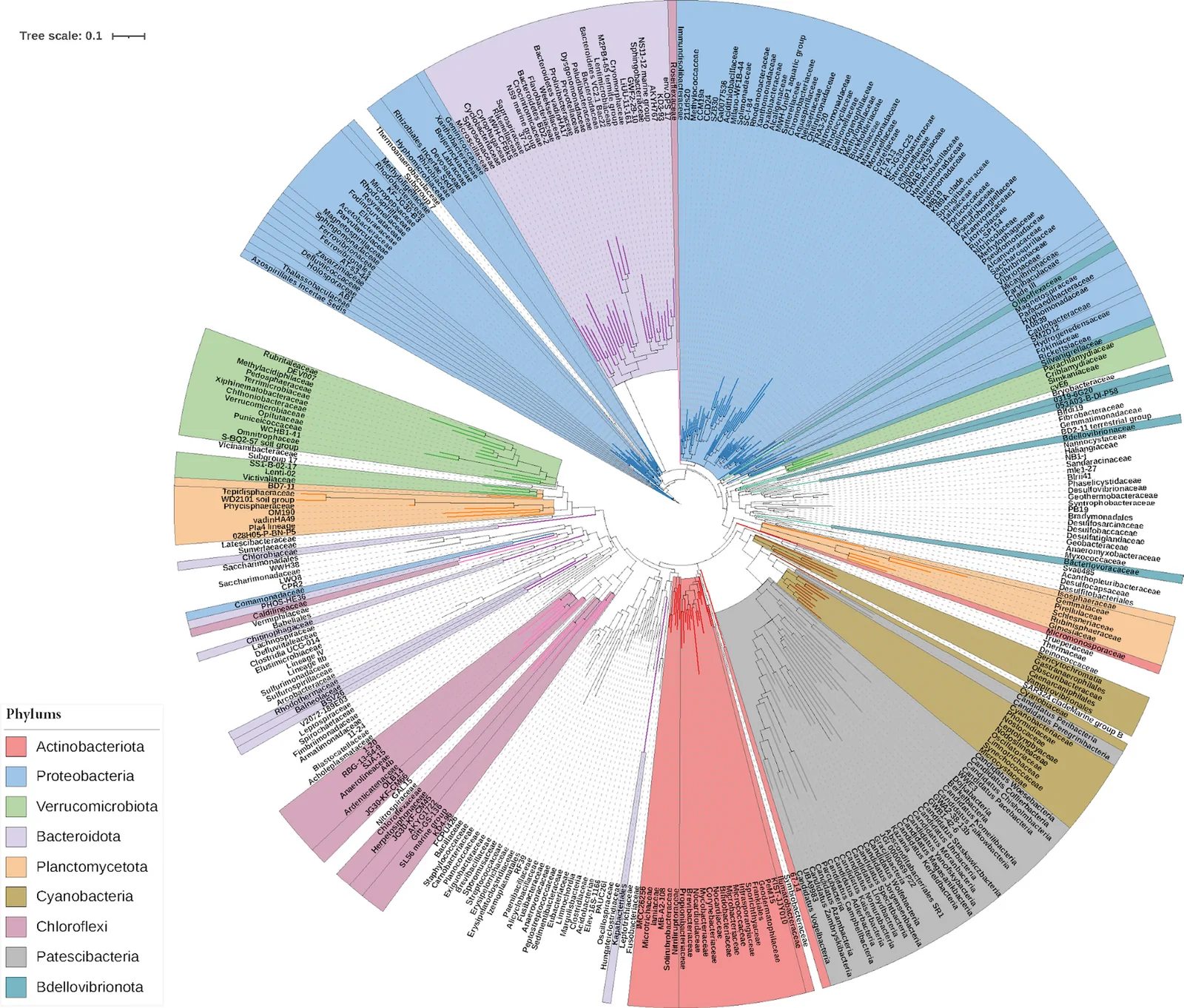
Circular phylogenetic tree that represented the taxonomic range and data of microbial communities in the Kitchener Drain. The tree illustrates the phylogenetic distribution of ASVs retrieved with the help of QIIME2 and DADA2 processing. Taxonomic family is denoted by a colored tip of the tree, and the seven most common phyla identified are Actinobacteriota, Proteobacteria, Verrucomicrobiota, Bacteroidota, Planctomycetota, Cyanobacteria, and Chloroflexi

A circular phylogenetic tree based on recovered ASVs revealed broad microbial diversity across seven major phyla, including Actinobacteriota, Proteobacteria, Verrucomicrobiota, Bacteroidota, Planctomycetota, Cyanobacteria, and Chloroflexi (Fig. 8). Proteobacteria showed high phylogenetic diversification, including environmentally and clinically relevant families such as Pseudomonadaceae, Vibrionaceae, Burkholderiaceae, Legionellaceae, Comamonadaceae, and Rhodobacteraceae. The eDNA analysis detected ASVs assigned to Burkholderiaceae and Legionellaceae; these DNA-based detections do not establish organism viability or explain their non-recovery from the intestinal-culture dataset.

The Actinobacteriota lineage included several pollution- and disease-associated families, including Mycobacteriaceae, Micrococcaceae, Propionibacteriaceae, and Nocardiaceae, with *Mycobacterium-*associated ASVs detected across sites. In addition, families associated with wastewater contamination and fish infection, including Staphylococcaceae, Streptococcaceae, Bacillaceae, and Erysipelotrichaceae, were identified, with *Erysipelothrix*-associated ASVs present at all sampling locations. Bacteroidota accounted for 10.75% ± 4.30% of the microbial community, whereas Cyanobacteria and Firmicutes represented 1.81% ± 0.88% and 1.24% ± 0.23%, respectively. The presence of Cyanobacteria families, including Microcystaceae and Nostocaceae, suggested potential algae–bacteria interactions influencing water quality and ecological dynamics of *Oreochromis niloticus* populations. Overall, phylogenetic profiling supported the integration of eDNA and remote sensing approaches for monitoring microbial pollution in drain (Fig. 8).

### Detection of different pathogenic bacteria in gut of the collected tilapia samples (CFU/g) using selective media

**Fig. 9:**
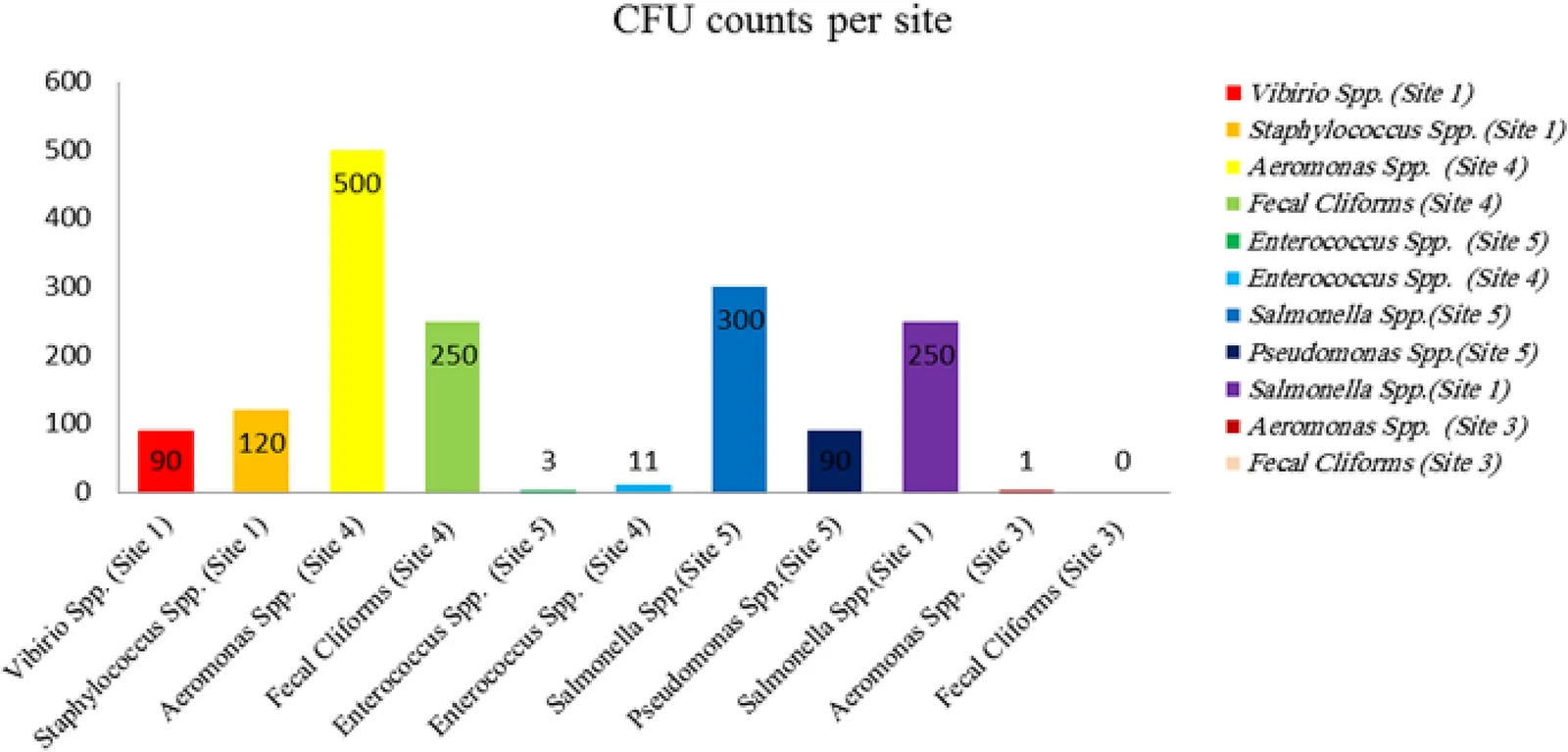
The microbial gut load using traditional culture techniques

Culture-dependent analysis using selective media revealed considerable variation in intestinal bacterial loads among collected *Oreochromis niloticus* samples, expressed as CFU g⁻^1^ (Fig. 9, Table 4). *Aeromonas* showed the highest abundance, exceeding 500 CFU g⁻^1^ in sample 4, whereas only 1 CFU g⁻^1^ was detected in sample 3. Moderate counts of presumptive *Salmonella* spp. were recorded, reaching 300 CFU g⁻^1^ in sample 5 and 250 CFU g⁻^1^ in sample 1. Fecal coliforms detected on M-FC agar reached 250 CFU g⁻^1^ in sample 4 but were absent in sample 3. Lower bacterial counts were observed for Staphylococcus (120 CFU g⁻^1^), *Vibrio* and *Pseudomonas* (90 CFU g⁻^1^ each), while *Enterococcus* showed the lowest abundance, ranging from 3–11 CFU g⁻^1^ (Fig. 9, Table 4).

**Table 4:** Media-Based Enumeration of Culturable Bacteria from Tilapia Gut Samples

Sample Site	Medium	Bacterial Count (CFU g⁻^1^)	Presumptive Identification	Representative isolates ID	VITEK®2 Identification Probability
1	Mannitol Salt Agar	120	*Staphylococcus* spp.	1 MA	*Staphylococcus aureus* 98% Probability
2	TCBS Agar	90	*Vibrio* spp.	2 TC	*Vibrio cholera* 98% Probability
3	Aeromonas Base Agar	1	*Aeromonas* spp.	3 AE	NA
4	Aeromonas Base Agar	> 500	*Aeromonas* spp.	4 AE	*Aeromonas hydrophila/punctata* (*A. caviae*) 99% Probability
4	M-FC Agar	250	Fecal coliforms* (E. coli)*	4 MF	*E. coli* 99% Probability
5	Slanetz & Bartley Agar	3	*Enterococcus* spp.	5 SL	NA
4	Slanetz & Bartley Agar	11	*Enterococcus* spp.	4 SLZ	NA
5	Bismuth Sulphite Agar	300	*Salmonella* spp.	5 BI	NA
5	Pseudomonas Agar	90	*Pseudomonas* spp.		*Pseudomonas aeruginosa* 98% Probability
1	Bismuth Sulphite Agar	250	*Salmonella* spp.		NA
3	M-FC Agar	0	Fecal coliforms		NA

NA: VITEK 2 identification was not performed for the corresponding isolateD

Identification of purified isolates using the VITEK 2 system confirmed the presence of several pathogenic and enteric bacteria within the intestinal microbiota, with *Aeromonas hydrophila* representing the predominant cultured species.

Water eDNA metabarcoding and culture-based analysis of Nile tilapia intestinal samples detected partially overlapping bacterial taxa across the five sampling sites. Because the two approaches were applied to different sample matrices, also their results were verified qualitatively for its presence in the gut, and no direct comparison of detection rates or microbial diversity was performed. This study tries to evaluate whether environmental DNA (eDNA) metabarcoding can provide a broader DNA-based profile of pathogen-associated taxa in the sampled water than culture-based examination of fish intestines alone; some taxa detected in the water by eDNA were qualitatively verified in the gut of fish using traditional methods. This was intended to assess the complementarity of the two approaches rather than their analytical performance [49].

*Aeromonas* was detected by both approaches, showing taxonomic overlap between the water-eDNA and intestinal-culture datasets. In contrast, eDNA metabarcoding detected additional pathogen-associated genera that were not observed in the intestinal-culture dataset, providing a broader DNA-based inventory for targeted follow-up. However, detection of bacterial DNA in water should not be interpreted as evidence of intestinal colonization or active infection [50, 51]**.** The detection of pathogen-associated bacterial DNA by 16S rRNA eDNA metabarcoding indicates the presence of bacterial genetic material in the sampled water but does not establish bacterial viability, infectivity, active infection, or pathogenicity.

### Phylogenetic relationships of pathogen-associated ASVs

**Fig. 10:**
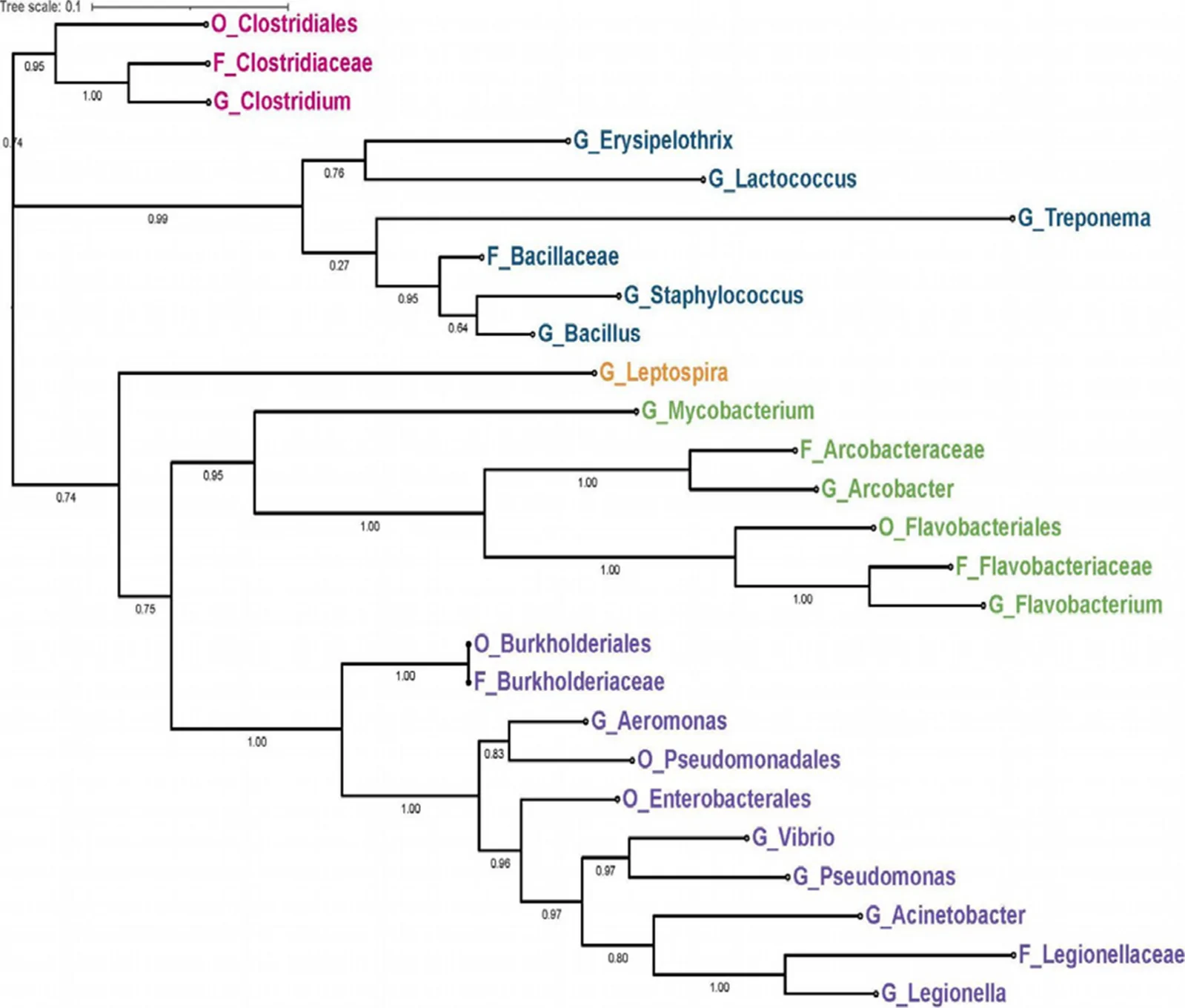
A reference phylogenetic tree of 26 pathogen-associated taxa detected across the five sampling sites, showing evolutionary relationships based on 16S rRNA (V3–V4) sequences. Taxa are labeled by rank (genus, family, order), and node support is indicated by bootstrap values (1,000 replicates), with a branch length scale of 0.1 substitutions per site

The reference phylogenetic tree (Fig. 10) encompassed 26 pathogen-associated taxa and demonstrated clustering patterns broadly consistent with established bacterial taxonomy. Firmicutes-associated taxa, including clostridial groups, *Erysipelothrix*, *Lactococcus*, and *Bacillus*, grouped within the same broad region of the tree, while Proteobacteria-associated taxa formed several distinct clusters in the lower portion, with *Pseudomonas*, *Acinetobacter*, and *Legionella* grouping together, Burkholderiales and Burkholderiaceae forming a separate well-supported branch, and Flavobacteriales and Arcobacteraceae each recovering their expected monophyletic groupings. Taxa with more divergent evolutionary histories, including *Treponema*, *Leptospira*, and *Mycobacterium*, were positioned outside the main Firmicutes and Proteobacteria clusters. One internal node involving Gram-negative aquatic taxa exhibited relatively low bootstrap support, suggesting limited phylogenetic resolution in this region, likely attributable to the conserved nature of the 16S rRNA marker. Overall, the topology was broadly consistent with the database-based taxonomic assignments, with most lineages clustering in accordance with their expected genus-, family-, or order-level affiliations.

**Fig. 11:**
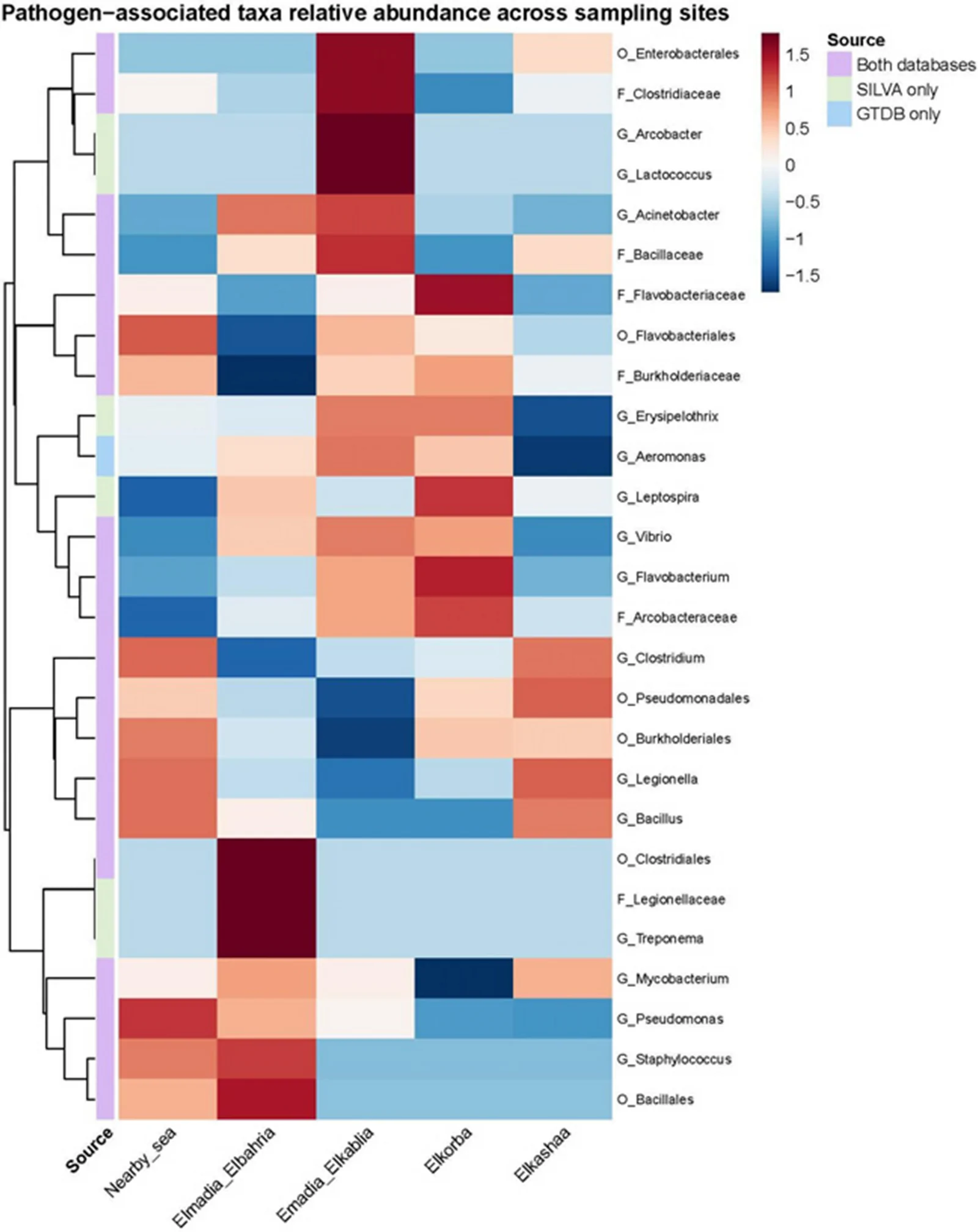
The heatmap shows relative abundance patterns of 32 pathogen-associated taxa across five sampling sites using combined SILVA 138 and GTDB r214 data. Values were log_10_(x + 0.01) transformed and Z-score normalized per taxon to highlight relative enrichment and depletion. Taxa were clustered using Euclidean distance with complete linkage, while sites were arranged in geographic order

Cross-database comparison of the QIIME 2 ASV table identified 256 pathogen-associated ASVs across 32 genera following union integration of SILVA database (138) and GTDB (r214). Of these, GTDB detected 123 ASVs and SILVA detected 205, with 72 ASVs shared between both databases; 51 were exclusive to GTDB and 133 to SILVA, indicating that reliance on a single database would have missed approximately 20–52% of pathogen-associated ASV diversity. Among shared ASVs, 58 (80.6%) showed full genus-level agreement, while 14 (19.4%) represented GTDB-resolved improvements over SILVA, and no taxonomic conflicts were observed. The pathogen-associated genera with the greatest read representation included *Mycobacterium* (detected across all five sites and all 32 ASVs), followed by Flavobacterium (enriched at Elkorba), *Clostridium* (ubiquitous), *Legionella* (notably abundant at Nearby Sea and Elkashaa), and *Erysipelothrix* (present across all sites) (Fig. 11).

At higher taxonomic resolution, Burkholderiales (389 total reads at Nearby Sea and Elkorba) represented a major pathogen-associated lineage, highlighting unresolved genus-level diversity within both databases and indicating the need for further taxonomic refinement. Taxa are annotated by database origin (SILVA-only, GTDB-only, or shared), and colors indicate relative enrichment (blue) or depletion (red), with near-zero values reflecting absence or minimal abundance. Total reads assigned to pathogen-associated taxa per site were 9,580 (Elmadia Elbahria), 9,346 (Elkashaa), 6,093 (Nearby Sea), 4,771 (Elkorba), and 4,499 (Elmadia Elkablia), indicating spatial variation in pathogen-associated read abundance (Fig. 11).

### Multivariate analysis of environmental variables and microbial communities

**Fig. 12:**
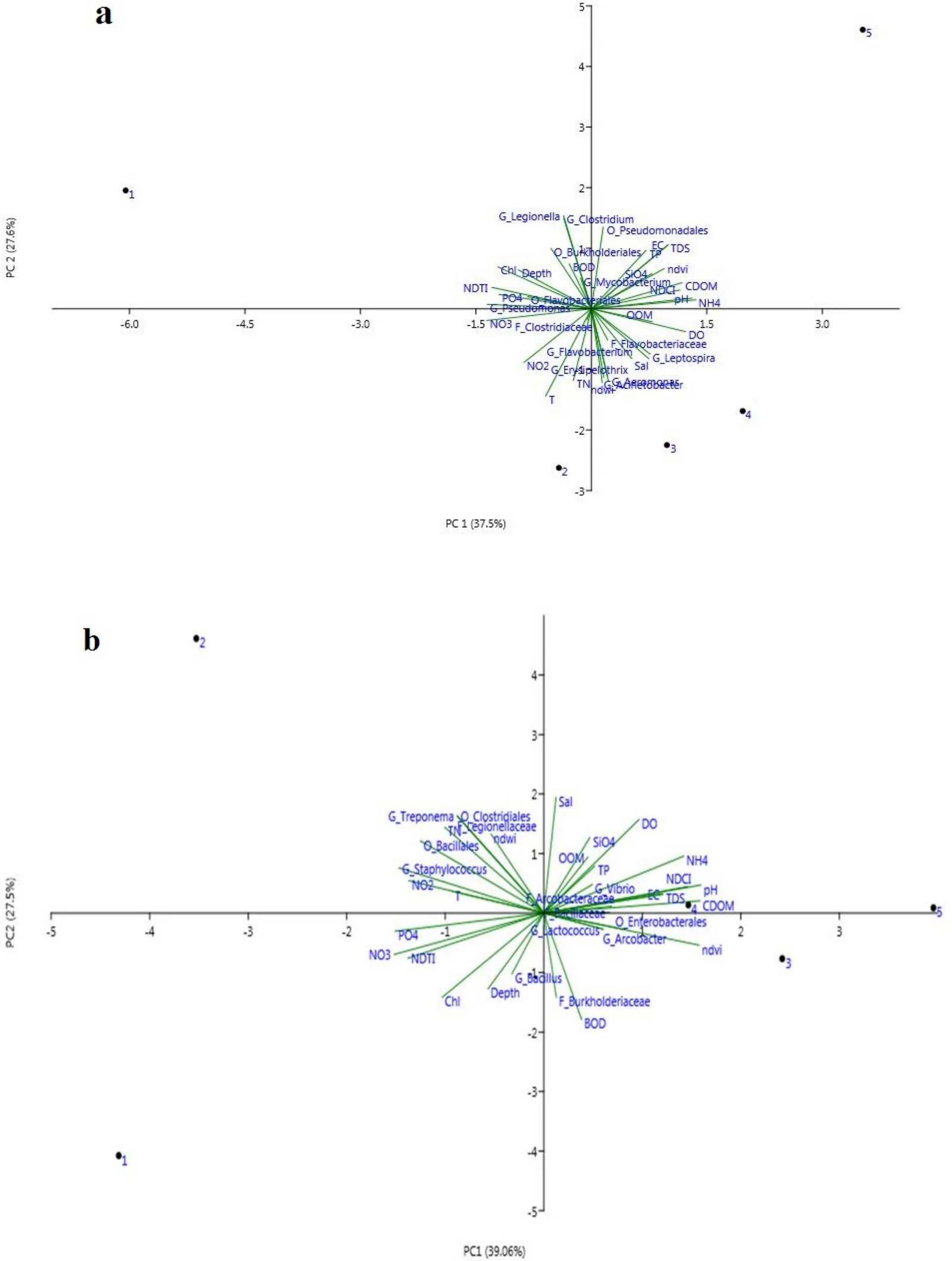
Principal Component Analysis (PCA) of environmental variables (EC, TDS, Sal, pH, T, Depth, DO, OOM, BOD, PO_4_, NH_4_, NO_2_, NO_3_, SiO_4_, TN, TP and Chl), spectral indices (NDWI, NDVI, NDTI, NDCI and CDOM) and microbial taxa across sampling sites; **a** *G_Mycobacterium*, *G_Flavobacterium*, O_Pseudomonadales, O_Burkholderiales, G_Pseudomonas, G_Legionella, *G_Acinetobacter*, *G_Clostridium, G_Erysipelothrix*, F_Flavobacteriaceae, O_Flavobacteriales, F_Clostridiaceae, *G_Leptospira* and *G_Aeromonas* (PC1 = 37.5% and PC2 = 27.6%). **b** *G_Bacillus*, F_Burkholderiaceae, F_Bacillaceae, G_Vibrio, F_Arcobacteraceae, *G_Staphylococcus*, O_Bacillales, O_Enterobacterales, O_Clostridiales, *G_Arcobacter*, *G_Lactococcus*, F_Legionellaceae and *G_Treponema* within water quality parameters (PC1 = 39.06% and PC2 = 27.5%)

Principal component analysis (PCA) provides strong statistical analysis for assessing relationships among multiple variables. From PCA (Fig. 12a), it is clear that PC1 and PC2 explained 65.1%. While PC3 and PC4 explained 34.8% from data. PC1 was characterized by positive loadings from NH_4_, NDCI, CDOM, pH and DO (that’s associated with site 5) and negative loadings from NO_3_, PO_4_, Chlorophyll, NDTI correlated with *G_Pseudomonas* (related with site 1). PC2 showed strong positive associations with EC, TDS, O_Pseudomonadales, *G_Legionella* and *G_Clostridium*. This component was negatively loaded by *G_Erysipelothrix* and *G_Aeromonas*. PC3 showed positive loading from salinity, *G_Mycobacterium*, NO_2_ and SiO_4_ and negative loadings from BOD and *G_Flavobacterium*. There is variability in the distribution of spectral indices and environmental parameters with the sampling sites.

From PCA (Fig. 12b), PCA 1 to 4 explained 39.06, 27.5, 20.45 and 12.97% from data, respectively. PC1 and PC2 together explained nearly 66.57% from data. PC1 was positively associated with NDVI, NDCI, CDOM, pH, NH_4_, EC and TDS. It is negatively loaded by NDTI, PO_4_, NO_2_, NO_3_, *G_Staphylococcus* and *O_Bacillales*. PC2 was positively associated and showed strong positive association with NDWI, salinity, SiO_4_, TN, and *O_Clostridiales, F_Legionellaceae* and *G_Treponema*. PC2 also showed strong negative association with depth, BOD and Chlorophyll and *F_Burkholderiaceae*. PC3 explained 20.45% of the total variance; it is high positively associated with temperature, *G_Vibrio*, *G_Arcobacter*, *F_Bacillaceae, G_Lactococcus F_Arcobacteraceae* and *O_Enterobacterales*, and negatively associated with SiO_4_, TP, and *G_Bacillus.*

### Correlation analysis between environmental variables and pathogen-associated taxa

To evaluate potential associations between microbial community composition and physicochemical water quality, Spearman rank correlation coefficients (ρ) were calculated between the relative abundances of 27 bacterial taxa and 13 physicochemical parameters: pH, temperature (Temp), dissolved oxygen (DO), biological oxygen demand (BOD), ammonium (NH_4_), nitrite (NO_2_), nitrate (NO_3_), phosphate (PO_4_), oxidizable organic matter (OOM), silicate (SiO_4_), total nitrogen (TN), total phosphorus (TP), and chlorophyll-a (Chl) across the five sampling sites in Kitchener Drain (n = 5; Table 5; full correlation matrix in Table S3). This resulted in 351 taxon–parameter comparisons. Because the number of comparisons was large relative to the number of sampling sites, exact two-sided permutation probabilities were calculated for each Spearman correlation coefficient using the exact null distribution for n = 5 [47]. Two perfect correlations were identified in the correlation matrix. G_Pseudomonas showed a perfect positive correlation with nitrate (ρ = + 1.00), whereas G_Acinetobacter showed a perfect negative correlation with chlorophyll-a (ρ = − 1.00). Both associations were nominally significant before multiple-testing correction (uncorrected p = 0.017). However, neither association remained statistically significant after correction for multiple comparisons. Given the small number of sampling sites and the large number of taxon–parameter comparisons, these perfect correlations were therefore interpreted cautiously as exploratory associations rather than evidence of environmental drivers or causal relationships.

**Table 5:** Spearman rank correlations between pathogen-associated taxa and physicochemical parameters across five sites in Kitchener Drain (n = 5)

Pathogen-associated taxon	Spearman Correlations (r value)
*G_Mycobacterium*	DO (r = + 0.67, p = 0.350); PO4 (r = + 0.67, p = 0.350); TP (r = + 0.60, p = 0.350)
*G_Flavobacterium*	PO4 (r = − 0.87, p = 0.133); Chl (r = − 0.70, p = 0.233); TN (r = + 0.60, p = 0.350); OOM (r = + 0.53, p = 0.450)
*G_Pseudomonas*	NO3 (r = + 1.00, p = 0.017); pH (r = − 0.90, p = 0.083); NH4 (r = − 0.90, p = 0.083)
*O_Pseudomonadales*	Chl (r = + 0.90, p = 0.083); Temp (r = − 0.87, p = 0.133)
*G_Acinetobacter*	Chl (r = −1, p = 0.017); TN (r = + 0.60, p = 0.350); BOD (r = −0.80, p = 0.133)
*Erysipelothrix piscisicarius*	BOD (r = + 0.90, p = 0.083); Chl (r = + 0.90, p = 0.083)
*G_Legionella*	Chl (r = + 0.80, p = 0.133); TP (r = + 0.70, p = 0.233)
*G_Clostridium*	BOD (r = + 0.90, p = 0.083); Chl (r = + 0.90, p = 0.083)
*G_Erysipelothrix*	TP (r = − 0.90, p = 0.083); SiO4 (r = − 0.70, p = 0.233); DO (r = − 0.56, p = 0.450)
F_Flavobacteriaceae	DO (r = − 0.87, p = 0.133); BOD (r = + 0.80, p = 0.133)
*O_Flavobacteriales*	SiO4 (r = − 0.90, p = 0.083); DO (r = − 0.87, p = 0.133); TP (r = − 0.80, p = 0.133)
F_Clostridiaceae	OOM (r = − 0.95, p = 0.083); TP (r = − 0.60, p = 0.350)
*G_Leptospira*	OOM (r = + 0.95, p = 0.083); SiO4 (r = + 0.60, p = 0.350)
*G_Aeromonas*	Chl (r = − 0.80, p = 0.133); TP (r = − 0.70, p = 0.233)
*G_Bacillus*	PO4 (r = + 0.89, p = 0.133); Chl (r = + 0.82, p = 0.133)
F_Burkholderiaceae	DO (r = − 0.87, p = 0.133); BOD (r = + 0.80, p = 0.133)
*G_Vibrio*	Chl (r = − 0.87, p = 0.133); PO4 (r = − 0.79, p = 0.233)
F_Arcobacteraceae	PO4 (r = − 0.87, p = 0.133); Chl (r = − 0.70, p = 0.233)
*G_Staphylococcus*	NO2 (r = + 0.89, p = 0.133); PO4 (r = + 0.80, p = 0.133); pH (r = − 0.78, p = 0.233)
*G_Treponema*	Temp (r = + 0.73, p = 0.233); BOD (r = − 0.71, p = 0.233)
F_Legionellaceae	Temp (r = + 0.73, p = 0.233); BOD (r = − 0.71, p = 0.233); NO2 (r = + 0.71, p = 0.233)

Note: *DO* dissolved oxygen, *OOM* oxidizable organic matter, *BOD* biological oxygen demand, *Chl* chlorophyll-a, *TN* total nitrogen, *TP* total phosphorus, *VBNC* viable but non-culturable

Several additional correlations showed relatively large effect sizes (|ρ|≥ 0.85), although none remained statistically significant after multiple-testing correction. These included negative associations of F_Clostridiaceae with OOM (ρ = − 0.95), G_Aeromonas with TP (ρ = − 0.80), G_Leptospira with PO_4_ (ρ = + 0.95), G_Flavobacterium with PO_4_ (ρ = − 0.87), F_Arcobacteraceae with PO_4_ (ρ = − 0.87), O_Flavobacteriales with SiO_4_ (ρ = − 0.90) and DO (ρ = − 0.87), F_Flavobacteriaceae and F_Burkholderiaceae with DO (ρ = − 0.87), and G_Staphylococcus with NO_2_ (ρ = + 0.89). Other relatively strong associations included positive correlations of G_Clostridium with BOD (ρ = + 0.90), O_Pseudomonadales with TP (ρ = + 0.90), and Piscisicarius with BOD and TP (ρ = + 0.90). These patterns indicate potential spatial co-variation between microbial taxa and environmental conditions but should be considered exploratory because of the limited number of sampling sites.

Overall, the correlation analysis did not provide statistically significant evidence that any individual physicochemical parameter was a definitive environmental driver of the observed microbial distributions after multiple-testing correction.

### Histopathological analysis

**Fig. 13:**
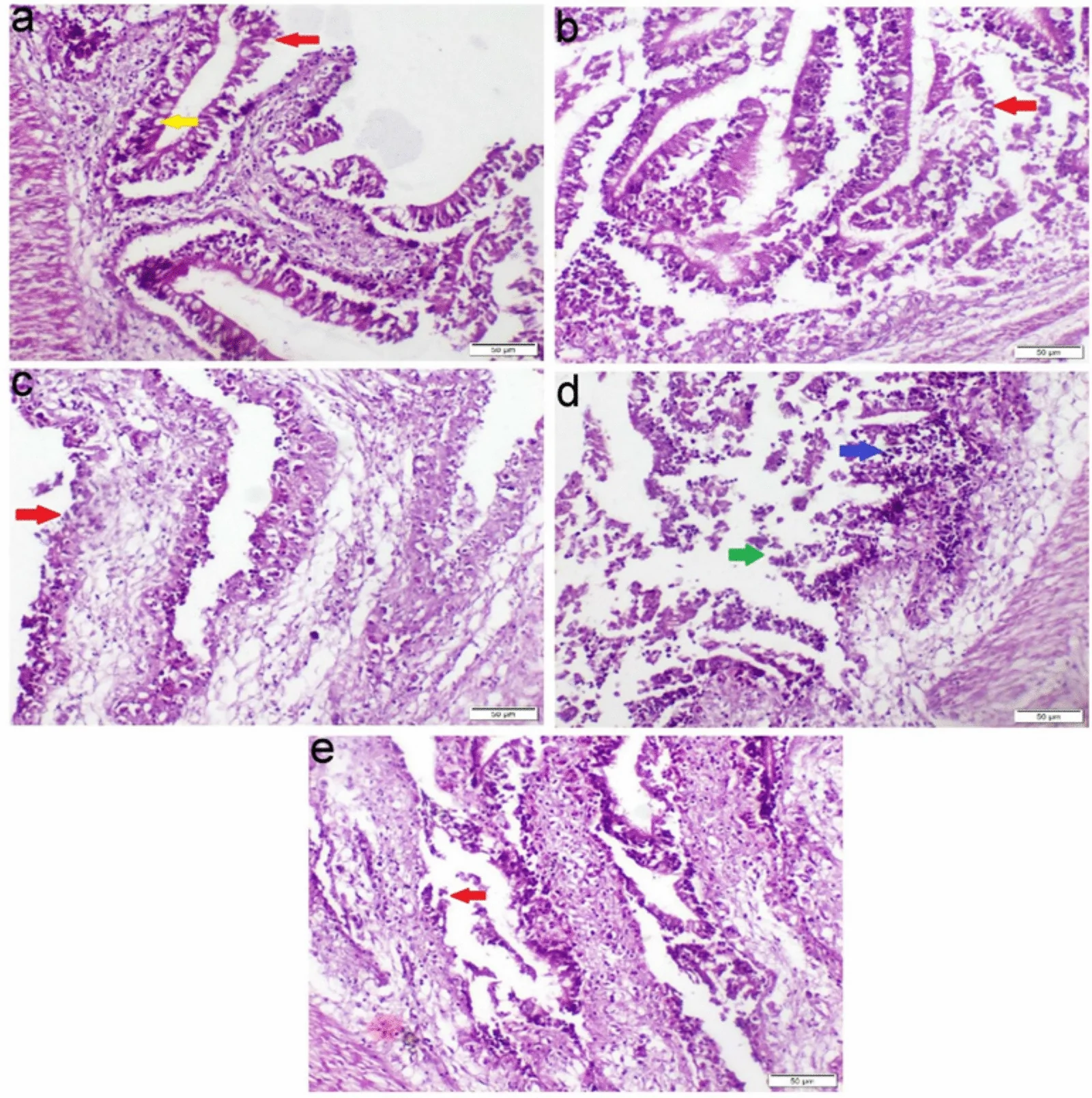
Photomicrographs of intestinal sections from various sampling sites are shown: **a** Nearby Sea; **b** Elmadia Elbahria; **c** Elmadia Elkablia; **d** Elkorba; and **e** Elkashaa. Observed lesions include intestinal necrosis (red arrows), infiltration of mononuclear inflammatory cells (blue arrows), goblet cell hyperplasia (yellow arrows), and complete loss of intestinal villi (green arrow). All sections were stained with H&E (scale bar = 50 μm)

**Fig. 14:**
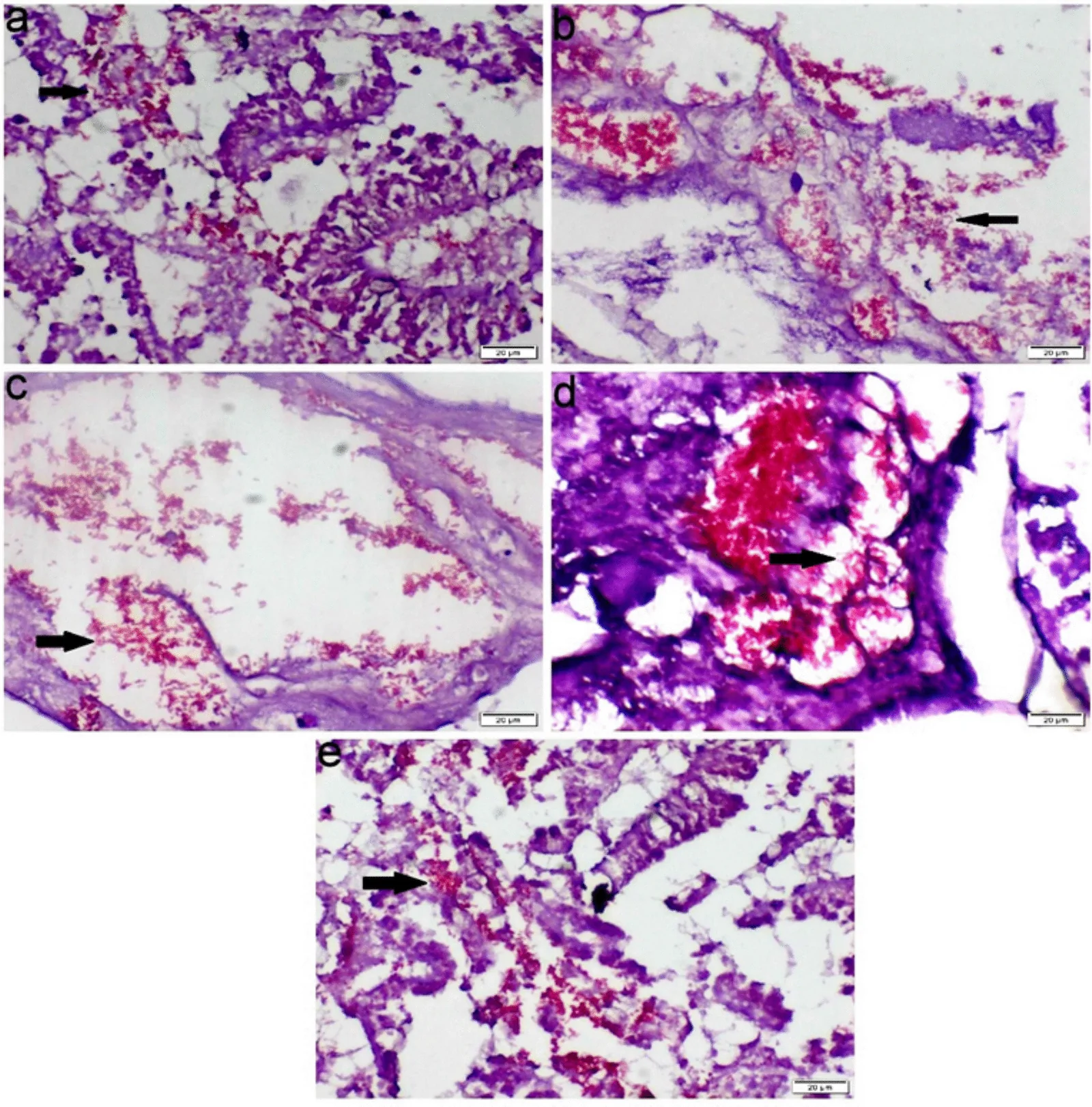
Photomicrographs of intestinal sections from various sampling sites are shown: **a** Nearby Sea; **b** Elmadia Elbahria; **c** Elmadia Elkablia; **d** Elkorba; and **e** Elkashaa. All sections were stained with the modified Gram stain (scale bar = 20 μm) and reveal abundant Gram-negative, rod-shaped bacteria within the intestinal tissue (black arrow)

Histological analysis of intestinal tissues from fish collected at the sampling sites revealed epithelial necrosis, with sloughed necrotic debris in the intestinal lumen and noticeable goblet cell hyperplasia. The Elkorba site exhibited the most severe lesions, characterized by complete villous necrosis and marked infiltration of mononuclear inflammatory cells (Fig. 13). Modified Gram staining demonstrated abundant Gram-negative, rod-shaped bacteria distributed throughout the intestinal tissue, with a large number recorded in the Elkorba site (Fig. 14).

Semi-quantitative lesion scores showed clear variation in intestinal histopathological alterations among the five sampling sites (Table 6). Fish collected from Nearby Sea and Elmadia Elbahria exhibited predominantly mild lesions, mainly represented by focal mucosal sloughing. In contrast, fish collected from Elkorba showed the most severe histopathological alterations such as extensive mucosal necrosis, collapse of the intestinal villi and marked inflammatory cell infiltration. Elkashaa exhibited moderate to severe lesions.

**Table 6:** Semi-quantitative histopathological lesion scores (mean ± SD) in the intestine of Nile tilapia (*Oreochromis niloticus*) collected from five sampling sites along Kitchener Drain

Site	Epithelial Necrosis	Inflammatory Infiltration	Goblet Cell Hyperplasia	Villous Atrophy/Degeneration	Mean Site Index	Overall Pathology Severity
Nearby Sea	0.33 ± 0.58	0.67 ± 0.58	0.33 ± 0.58	0.00 ± 0.00	0.33 ± 0.28	Normal/Minimal
Elmadia Elbahria	1.00 ± 0.00	0.67 ± 0.58	1.00 ± 0.00	0.67 ± 0.58	0.83 ± 0.24	Mild
Elmadia Elkablia	1.67 ± 0.58	2.00 ± 0.00	1.67 ± 0.58	1.67 ± 0.58	1.75 ± 0.25	Moderate
Elkorba	3.67 ± 0.58	3.67 ± 0.58	3.33 ± 0.58	3.67 ± 0.58	3.58 ± 0.29	Severe/Extensive
Elkashaa	2.33 ± 0.58	2.67 ± 0.58	2.33 ± 0.58	2.67 ± 0.58	2.50 ± 0.20	Marked/Severe

Note: Lesion severity was scored according to the semi-quantitative scoring system of Bernet et al. [44] using a five-point scale (0 = absent, 1 = mild, 2 = moderate, 3 = marked, and 4 = severe). Values are presented as mean ± SD (n = 3 fish per site). The Mean Site Index represents the arithmetic mean of the evaluated lesion scores for each sampling site

## Discussion

The results of this study demonstrate that Kitchener Drain is a highly eutrophic freshwater system strongly influenced by continuous anthropogenic loading from agricultural runoff, municipal wastewater, and industrial discharges. The elevated concentrations of ammonium, nitrate, phosphate, total nitrogen, and chlorophyll-a, together with reduced dissolved oxygen at several sites, confirm advanced nutrient enrichment and ecosystem degradation. The particularly low dissolved oxygen recorded at the Nearby Sea site (2.14 mg/L) indicates localized hypoxic stress, which is expected to significantly influence microbial activity and fish physiological stability, consistent with previously reported eutrophic freshwater systems [52, 53].

The water-eDNA communities were dominated by Actinobacteriota (27.35 ± 6.19%), Proteobacteria/Pseudomonadota (26.73 ± 3.24%), Verrucomicrobiota (15.02 ± 4.96%), Bacteroidota (10.75 ± 4.30%), and Planctomycetota (6.89 ± 2.93%). A comparable predominance of Pseudomonadota, Actinobacteriota, and Bacteroidota was reported in planktonic communities from different Danube River water-body types [54]. However, that study also found that community structure differed by habitat and water-body type, illustrating that these widely distributed phyla are not specific indicators of pollution. Similarly, Liu et al. [53] found spatial and seasonal covariation among bacterial communities, water chemistry, and land use, but their analysis included ten rivers, multiple locations per river, four seasons, and quantified land-use data. The present results are therefore compatible with environmental heterogeneity but cannot attribute the observed community differences specifically to pollution.

Spatial diversity analyses showed that microbial community structure varied among sites in association with environmental heterogeneity. Higher alpha diversity at the Nearby Sea site (1,920 ASVs) may be consistent with hydrological mixing and increased environmental connectivity with marine-influenced waters, enhancing niche availability. In contrast, lower diversity at inland sites was observed alongside higher pollutant levels, although this does not demonstrate environmental filtering. Beta diversity results indicated that species turnover accounted for most of the observed community dissimilarity, indicating taxonomic replacement among sites through taxonomic replacement along environmental gradients rather than simple loss of taxa [55]. Zhang et al. [56] also documented spatially heterogeneous bacterial assemblages in a heavily polluted urban river, but environmental drivers were evaluated using a broader horizontal and vertical sampling design and explicit community-assembly analyses. Equivalent driver attribution is not possible with five unreplicated site observations.

The high diversity at the Nearby Sea may be consistent with mixing or greater environmental connectivity, but this remains a hypothesis rather than a demonstrated mechanism. Site identity in the present study was inseparable from position along the drain, salinity, flow conditions, residence time, potential tidal or marine influence, surrounding land use, and unmeasured point and diffuses discharges. In a study of 300 samples across a river-to-ocean gradient, Fortunato et al. [57] found that bacterial-community ordination was strongly correlated with salinity (ρ = − 0.83\rho = −0.83ρ = − 0.83) and depth (ρ = − 0.62\rho = −0.62ρ = − 0.62). Read et al. [58], using 23 sites in the Thames basin, found that downstream community succession was associated more strongly with water residence time than with the measured physicochemical variables. Hydrology may also affect the locality of the eDNA signal because river water can integrate DNA transported from upstream and adjacent terrestrial or aquatic sources [59]. These studies demonstrate that hydrological position and salinity provide credible alternative explanations for the apparent pollution gradient.

A key outcome of this study is spatial co-variation between environmental conditions and eDNA detection patterns of pathogen-associated taxa. The integration of SILVA and GTDB databases with eDNA metabarcoding broadened the taxonomic assignment coverage of pathogen-associated ASVs, assigning 256 ASVs to pathogen-associated taxa across 32 genera. This highlights differences in taxonomic coverage between the reference databases; it does not permit a direct sensitivity comparison with culture because different sample matrices were analyzed. SILVA assigned more ASVs to pathogen-associated taxa likely because of its broader 16S rRNA reference coverage, whereas GTDB added complementary phylogenomically refined assignments. Therefore, using both databases reduced reference-dependent bias and broadened the database-dependent taxonomic coverage of pathogen-associated taxa. Furthermore, 16S rRNA read data are compositional; an increase in the relative abundance of one taxon necessarily affects the relative proportions of other taxa, potentially influencing taxon–environment correlations [60]. Pathogen-associated taxa including *Mycobacterium*, *Legionella*, *Erysipelothrix*, and members of Burkholderiaceae were detected as DNA-based taxonomic assignments in the water-eDNA dataset but were not recovered from cultured fish-intestinal samples. Because the two approaches examined different sample matrices, this difference cannot be attributed to cultivation difficulty, analytical sensitivity, or host filtering.

Culture-based analysis confirmed the presence of viable pathogen-associated bacteria in Nile tilapia intestinal contents, indicating active microbial exposure consistent with the environmental contamination levels documented across sampling sites. The predominance of *Aeromonas hydrophila*/*A. caviae* in the most organically enriched site is consistent with its established role as an opportunistic pathogen thriving under conditions of high organic loading and compromised host immunity in freshwater fish [6, 61]. The recovery of presumptive *Salmonella* spp. at two sites, despite the absence of VITEK®2 confirmation, raises public health concerns given the zoonotic potential of this genus and the dual use of Kitchener Drain as both an aquaculture source and an irrigation waterway [62]. The co-occurrence of fecal coliforms and presumptive *Salmonella* spp. further reflects chronic wastewater input into the drain, consistent with the elevated oxidizable organic matter and nutrient concentrations documented in the physicochemical analysis.

Notably, *Aeromonas* was the only genus consistently detected by both culture-based and eDNA approaches, showing taxonomic overlap between the water-eDNA and intestinal-culture datasets. This finding supports previous evidence that *Aeromonas* can function as both a commensal and opportunistic pathogen depending on environmental conditions [7, 63].

Spatial microbial profiling (heatmap and phylogenetic analyses) revealed strong site-specific enrichment patterns. Elkashaa was characterized by higher abundance of *Mycobacterium* and *Nitrosomonas*, whereas Elkorba and Elmadia Elkablia showed enrichment of *Flavobacterium* and *Hydrogenophaga*.

The enrichment of *Mycobacterium* at Elkashaa, despite only non-significant correlations with the measured physicochemical parameters (DO: ρ = + 0.67; PO4: ρ = + 0.67; Table 5), suggests that no strong association with individual water-column variables was detected. Instead, this pattern may reflect the ecological versatility of aquatic mycobacteria, which can persist in protective microhabitats such as biofilms, sediments, and aquatic amoebae [64, 65]. Thus, the enrichment of *Mycobacterium* at Elkashaa may indicate localized sediment- or biofilm-associated persistence, although this interpretation requires targeted sediment and biofilm sampling in future studies.

Two perfect taxon–environment correlations were also observed: a positive association between G_Pseudomonas and nitrate (ρ = + 1.00) and a negative association between G_Acinetobacter and chlorophyll-a (ρ = − 1.00). Although both associations were nominally significant before multiple-testing correction (uncorrected p = 0.017), neither remained statistically significant after correction for multiple comparisons. These results should therefore be interpreted as exploratory spatial associations rather than evidence that nitrate or chlorophyll-a directly drives the distribution of these taxa.

These patterns reflect spatial variability in nutrient loading, organic matter accumulation, and hydrological conditions. The widespread occurrence of *Erysipelothrix* across all sites further shows broad spatial detection of *Erysipelothrix*-associated sequences during the single sampling event.

PCA was performed to understand the interactions between the physicochemical variables, microbiological parameters and spectral indices data from satellite images [66, 67]. Recent studies have demonstrated the predictive value of combining spectral indices with microbial datasets for waterborne pathogen modeling [19, 20, 68]. Authors used bioinformatics in surface water studies. The bacterial community patterns were associated with the physiochemical characteristics, chemical and biological contamination [69, 71]. Dominant microbial taxa are varied as a result of organic matter content in the environment [72].

The genera *Aeromonas* wasn’t showing more preference for location or the presence of organic matter [73], this is agreed with distribution in Kitchener drain. As, it was distributed along the drain, although the high concentration of OOM was concentrated more in the southern part of the studied area. It is obvious that algae and particulates are heavy colonized hotspots of bacterial activity especially in freshwater ecosystems. The organic matter and particles represent diverse niches that were exploited by various organisms [74]. In agricultural streams, microbial functions were correlated with more labile, protein-like DOM, PO_4_, and NO_3_, likely reflecting functional adaptation to labile DOM and higher nutrient concentrations. *Flavobacterium* was isolated from sewage lines and diseased fish. Organic compounds play a role in their predominance in the summer season [53, 75].

Sea surface temperature and salinity are important indicators for growth conditions. The preferred growth conditions of bacteria were warm water (> 15 ℃) with salinity degrees lower than 25 ppt seawater. Also, the tidal intrusion of coastal sea waters carrying plankton laden with *Vibrio* spp. in inland river systems [76, 77]. Those findings agree with our results as *Vibrio sp* was positively associated with PC3 in addition to temperature. The mycobacterium was abundant at locations with highly nutrient enrichment [64, 65]. *G_Pseudomonas* showed a reverse trend with ammonia as this species was known to assimilate ammonia–nitrogen directly [78].

Also the PCA discusses the correlation between sites, spectral indices and variables. For example in PCA (a), CDOM showed positive loading with PC1 and negatively with NDTI. Hong et al. [19] found similar patterns between variables such as pigments, organic carbon in water and turbidity with used remote sensing spectral indices in their study. In PCA (b), NDVI, NDCI, CDOM and NH_4_ were associated with sites 4 and 5 (these two sites characterized by high organic load). It could be interpreted that the high OOM was in site 4 and NH_4_ in site 5 impact on the reflectance of these indices. CDOM was an indicator of the impact of detritus wastes from drainage waters discharge. CDOM was impacted by many factors such as the soluble microbial metabolites in aquatic vegetation and the release from rivers and drains [79, 80]. Also some species such as *G_Staphylococcus* are associated and take similar loading with index of turbidity NDTI. Steadmon et al. [81] stated that *Staphylococcus aureus* in fresh, brackish and marine waters related positively with turbid water. Also, the observed association of *Vibrio* spp. with temperature and PC3(b) is consistent with these ecological dynamics. Similarly, in PC3(a) *Mycobacterium* spp. showed higher abundance in nutrient-enriched sites [64, 65], while the negative relationship between *Pseudomonas* spp. and ammonia from correlation analysis may be consistent with the reported capacity of some *Pseudomonas* spp. for ammonia utilization [78].

Ecologically, microbial distribution patterns reflect strong associations with organic matter availability and particulate substrates, which serve as key colonization niches in freshwater ecosystems [74]. The predominance of *Flavobacterium* in organic-rich environments aligns with its known association with sewage-impacted waters and diseased fish, particularly under warm conditions [53, 75]. Environmental factors such as temperature and salinity further modulate microbial structure, with warm, low-salinity conditions associated with bacterial community variation, while hydrological connectivity may contribute eDNA signals from marine-associated taxa such as *Vibrio* spp. into freshwater systems [76, 77].

Histopathological findings provide direct biological confirmation of the environmental and microbial impacts observed. Severely polluted sites, particularly Elkorba, exhibited marked intestinal pathology including epithelial necrosis, villous atrophy, goblet cell hyperplasia, inflammatory infiltration, and extensive Gram-negative bacterial colonization. These lesions are consistent with chronic bacterial enteritis and long-term exposure to degraded water quality [18]. The observed mucosal responses likely represent both protective (goblet cell proliferation) and degenerative (villous loss, epithelial damage) processes affecting intestinal function.

Finally, the detection of DNA assigned to pathogen-associated and potentially opportunistic taxa such as *Clostridium*, *Legionella*, *Mycobacterium*, and *Erysipelothrix* raises important environmental and public health concerns. These findings indicate that polluted freshwater systems may act as persistent reservoirs of pathogenic bacteria, posing risks to aquaculture productivity, fish health, and human exposure.

## Conclusion

This study demonstrates that eutrophication, organic pollution, and decrease of oxygen are factors illuminating shaping pathogen-associated taxa in Kitchener Drain. By integrating eDNA metabarcoding, remote sensing, conventional bacteriology, and fish histopathology, the study conducted spatial associations between environmental degradation, relative enrichment of eDNA reads assigned to pathogen-associated taxa, and fish health impairment and highlighted the need for targeted zoonotic-risk assessment. The results highlight the value of combining satellite-derived environmental indicators with molecular surveillance tools for screening of pathogen-associated eDNA signals and ecosystem health assessment in aquaculture-associated waters. Integration of spectral indices with water quality parameters explains the surrounding environment for those pathogens. For example NDTI can illustrate the distribution of turbidity and relation to pathogen species as *G_Pseudomonas* (PCA analysis as indication). eDNA plays an important role in inventory of many species (database for most pathogen-associated taxa in northern part of Kitchener drain), so aid in supporting the fisheries sector. Sites with high organic pollutants impacted badly on the health of fish. This appears in fish health; appearance of epithelial necrosis, inflammation, and presence of G-negative bacteria.

## Limitations

No filtration negative controls were included; therefore, potential contamination introduced during filtration cannot be excluded, and low-abundance taxonomic detections should be interpreted cautiously. Also 16S amplicon detection only indicates the presence of DNA and does not demonstrate cell viability, virulence, or host infection. Remote sensing data was applied to only indicate the case of environmental conditions associated with eDNA detection patterns of pathogen-associated taxa, but not as a predictive tool here.

## Supplementary Information

Supplementary Material 1.

## Data Availability

The datasets generated and/or analyzed during the current study are included in the manuscript and its supplementary material. The raw FASTQ files have been deposited in the NCBI Sequence Read Archive (SRA) under BioProject accession number PRJNA1489777.
