# Supplementary material for: Multi-approach integrating water quality variables, remote sensing, eDNA and histopathology for assessing the impact of microbial pollution on Nile Tilapia from Kitchener Drain, Northern Egypt
Source: BMC Microbiol. 2026 Sep 5;26:793. doi: 10.1186/s12866-026-05559-7 (PMC13555907; doi:10.1186/s12866-026-05559-7)
Supplement: Supplementary file 1 [file 12866_2026_5559_MOESM1_ESM.docx]

Supplementary material

Journal: BMC Microbiology

Title: Multi-approach integrating water quality variables, remote sensing, eDNA, and histopathology for assessing the impact of microbial pollution on Nile Tilapia from Kitchener Drain, Northern Egypt

Table S1: Relative abundance of pathogen-associated taxa along the study area

| **Taxon** | **Station** | | | | |
| --- | --- | --- | --- | --- | --- |
|  | **Nearby_sea** | **Elmadia_Elbahria** | **Emadia_Elkablia** | **Elkorba** | **Elkashaa** |
| ***G_Mycobacterium*** | 58.1979 | 69.7808 | 57.9907 | 31.7753 | 68.0184 |
| ***G_Flavobacterium*** | 3.1347 | 5.5741 | 18.7375 | 39.4257 | 3.6165 |
| **O_Pseudomonadales** | 9.7489 | 6.6806 | 4.3565 | 9.5368 | 12.4438 |
| ***E. Piscisicarius*** | 6.8275 | 4.2276 | 2.5561 | 5.743 | 5.6816 |
| ***G_Pseudomonas*** | 10.7336 | 5.5741 | 3.0451 | 1.0061 | 0.9844 |
| ***G_Legionella*** | 3.9062 | 1.19 | 0.5779 | 1.1528 | 4.1836 |
| ***G_Acinetobacter*** | 0.279 | 2.5261 | 3.4008 | 0.4402 | 0.3103 |
| ***G_Clostridium*** | 1.7397 | 0.6472 | 0.9558 | 1.027 | 1.7013 |
| ***G_Erysipelothrix*** | 0.9027 | 0.8038 | 2.4672 | 2.4523 | 0.2354 |
| **F_Flavobacteriaceae** | 1.0832 | 0.3862 | 1.0224 | 3.7938 | 0.4066 |
| **O_Flavobacteriales** | 1.395 | 0.4071 | 1.1114 | 0.9222 | 0.6527 |
| **F_Clostridiaceae** | 0.6729 | 0.6054 | 0.8891 | 0.545 | 0.6527 |
| ***G_Leptospira*** | 0.1641 | 0.4593 | 0.289 | 0.6917 | 0.3317 |
| ***G_Aeromonas*** | 0.1313 | 0.3236 | 0.978 | 0.4402 | 0 |
| ***G_Bacillus*** | 0.5908 | 0.1044 | 0 | 0 | 0.5243 |
| **F_Burkholderiaceae** | 0.3447 | 0 | 0.2667 | 0.4192 | 0.107 |
| **F_Bacillaceae** | 0 | 0.0939 | 0.5557 | 0 | 0.0963 |
| ***G_Vibrio*** | 0 | 0.1461 | 0.3334 | 0.2515 | 0 |
| **F_Arcobacteraceae** | 0 | 0.0418 | 0.1778 | 0.3773 | 0.0321 |
| ***G_Staphylococcus*** | 0.0985 | 0.167 | 0 | 0 | 0 |
| **O_Bacillales** |  | 0.1566 | 0 | 0 | 0 |
| **O_Enterobacterales** | 0 | 0 | 0.1111 | 0 | 0.0214 |
| **O_Clostridiales** | 0 | 0.0626 | 0 | 0 | 0 |
| ***G_Arcobacter*** | 0 | 0 | 0.1111 | 0 | 0 |
| ***G_Lactococcus*** | 0 | 0 | 0.0667 | 0 | 0 |
| **F_Legionellaceae** | 0 | 0.0209 | 0 | 0 | 0 |
| ***G_Treponema*** | 0 | 0.0209 | 0 | 0 | 0 |

Table S2: Extracted values of calculated spectral indices

| No | Name | Spectral Index | | | | |
| --- | --- | --- | --- | --- | --- | --- |
|  |  | NDWI | NDVI | NDTI | NDCI | CDOM |
| 1 | Nearby Sea | 0.031511 | -0.018514 | -0.013004 | -0.01873 | 0.972604 |
| 2 | Elmadia Elbahria | 0.046899 | -0.02424 | -0.022694 | 0.019605 | 1.036317 |
| 3 | Elmadia Elkablia | 0.033154 | -0.004393 | -0.028773 | 0.073506 | 1.210725 |
| 4 | Elkorba | 0.045715 | -0.008703 | -0.032496 | 0.022957 | 1.114087 |
| 5 | Elkashaa | 0.028613 | 8.55E-05 | -0.02875 | 0.086206 | 1.265991 |

Table S3. Full Spearman correlation matrix between microbial taxa and environmental parameters in Kitchener Drain.

| **Spearman correlation** |  |  |  |  |  | |  | | **Water quality parameters** | | | | |  |  |
| --- | --- | --- | --- | --- | --- | --- | --- | --- | --- | --- | --- | --- | --- | --- | --- |
|  | **PH** | **Temp** | **DO** | **BOD** | **NH4** | **NO2** | | **NO3** | | **PO4** | **OOM** | **SiO4** | **TN** | **TP** | **chl** |
| ***G_Mycobacterium*** | -0.1 | 0.21 | 0.67 | -0.5 | -0.1 | 0.4 | | 0.2 | | 0.67 | -0.11 | 0.5 | 0 | 0.6 | 0.1 |
| ***G_Flavobacterium*** | 0.3 | 0.31 | 0.05 | -0.3 | 0.3 | -0.2 | | -0.4 | | -0.87 | 0.53 | 0.1 | 0.6 | -0.2 | -0.7 |
| **O_Pseudomonadales** | 0.1 | -0.87 | -0.05 | 0.6 | 0.1 | -0.5 | | -0.3 | | 0.41 | 0.21 | 0.3 | -0.7 | 0.6 | 0.9 |
| **E. Piscisicarius** | -0.5 | -0.56 | -0.67 | 0.9 | -0.5 | -0.1 | | 0.2 | | 0.46 | 0.16 | -0.3 | -0.3 | 0.1 | 0.9 |
| ***G_Pseudomonas*** | -0.9 | 0.56 | -0.46 | 0.1 | -0.9 | 0.9 | | 1 | | 0.67 | -0.58 | -0.7 | 0.3 | -0.5 | 0.1 |
| ***G_Legionella*** | 0 | -0.61 | 0.21 | 0.3 | 0 | -0.2 | | -0.1 | | 0.67 | 0.05 | 0.4 | -0.6 | 0.7 | 0.8 |
| ***G_Acinetobacter*** | 0.3 | 0.72 | 0.41 | -0.8 | 0.3 | 0.2 | | -0.1 | | -0.62 | 0 | 0.1 | 0.6 | -0.3 | -1 |
| ***G_Clostridium*** | -0.1 | -0.82 | -0.56 | 0.9 | -0.1 | -0.4 | | 0 | | 0.36 | -0.21 | -0.3 | -0.8 | 0 | 0.9 |
| ***G_Erysipelothrix*** | -0.1 | 0.36 | -0.56 | 0.1 | -0.1 | 0.1 | | 0.2 | | -0.56 | -0.26 | -0.7 | 0.3 | -0.9 | -0.5 |
| **F_Flavobacteriaceae** | -0.2 | -0.36 | -0.87 | 0.8 | -0.2 | -0.3 | | 0 | | -0.31 | 0.11 | -0.6 | -0.1 | -0.5 | 0.3 |
| **O_Flavobacteriales** | -0.3 | -0.21 | -0.87 | 0.7 | -0.3 | 0 | | 0.4 | | 0.051 | -0.63 | -0.9 | -0.4 | -0.8 | 0.3 |
| **F_Clostridiaceae** | 0.1 | 0.05 | -0.15 | 0 | 0.1 | 0.1 | | 0.3 | | 0.1 | -0.95 | -0.5 | -0.5 | -0.6 | -0.1 |
| ***G_Leptospira*** | 0.2 | 0.1 | 0.36 | -0.3 | 0.2 | -0.2 | | -0.5 | | -0.46 | 0.95 | 0.6 | 0.6 | 0.5 | -0.3 |
| ***G_Aeromonas*** | -0.36 | 0.62 | -0.21 | -0.3 | 0 | 0.2 | | 0.1 | | -0.67 | -0.05 | -0.4 | 0.6 | -0.7 | -0.8 |
| ***G_Bacillus*** | 0 | -0.39 | -0.11 | 0.41 | -0.36 | 0.15 | | 0.36 | | 0.89 | -0.35 | -0.05 | -0.56 | 0.31 | 0.82 |
| **F_Burkholderiaceae** | -0.2 | -0.36 | -0.87 | 0.8 | -0.2 | -0.3 | | 0 | | -0.31 | 0.11 | -0.6 | -0.1 | -0.5 | 0.3 |
| **F_Bacillaceae** | 0.72 | 0.16 | 0.63 | -0.67 | 0.72 | -0.2 | | -0.36 | | -0.39 | -0.35 | 0.31 | -0.21 | -0.05 | -0.62 |
| ***G_Vibrio*** | 0.21 | 0.55 | -0.03 | -0.41 | 0.21 | 0.05 | | -0.1 | | -0.79 | 0.05 | -0.21 | 0.56 | -0.56 | -0.87 |
| **F_Arcobacteraceae** | 0.3- | 0.31 | 0.05 | -0.3 | 0.3 | -0.2 | | -0.4 | | -0.87 | 0.53 | 0.1 | 0.6 | -0.2 | -0.7 |
| ***G_Staphylococcus*** | -0.78 | 0.57 | 0.06 | -0.22 | -0.78 | 0.89 | | 0.78 | | 0.8 | -0.18 | -0.11 | 0.45 | 0.11 | 0.11 |
| **O_Bacillales** | -0.77 | 0.77 | 0.54 | -0.77 | -0.77 | 0.77 | | 0.77 | | 0.82 | 0 | 0.26 | 0.77 | 0.26 | -0.26 |
| **O_Enterobacterales** | 0.78 | -0.11 | 0.34 | -0.34 | 0.78 | -0.45 | | -0.45 | | -0.52 | -0.41 | 0.11 | -0.45 | -0.22 | -0.45 |
| **O_Clostridiales** | -0.35 | 0.73 | 0.54 | -0.71 | -0.35 | 0.71 | | 0.35 | | 0.36 | 0.19 | 0.35 | 0.71 | 0.35 | -0.35 |
| ***G_Arcobacter*** | 0.35 | 0.36 | 0 | -0.35 | 0.35 | 0 | | 0 | | -0.54 | -0.56 | -0.35 | 0 | -0.71 | -0.71 |
| ***G_Lactococcus*** | 0.35 | 0.36 | 0 | -0.35 | 0.35 | 0 | | 0 | | -0.54 | -0.56 | -0.35 | 0 | -0.71 | -0.71 |
| **F_Legionellaceae** | -0.35 | 0.73 | 0.54 | -0.71 | -0.35 | 0.71 | | 0.35 | | 0.36 | 0.19 | 0.35 | 0.71 | 0.35 | -0.35 |
| ***G_Treponema*** | -0.35 | 0.73 | 0.54 | -0.71 | -0.35 | 0.71 | | 0.35 | | 0.36 | 0.19 | 0.35 | 0.71 | 0.35 | -0.35 |
